# Maintenance therapy for cytogenetically high-risk multiple myeloma: landscape in the era of novel drugs

**DOI:** 10.1007/s10238-024-01445-6

**Published:** 2024-08-06

**Authors:** Xinyuan Gu, Wenjiao Tang, Li Zhang, Yuhuan Zheng, Ling Pan, Ting Niu

**Affiliations:** 1 Department of Hematology, Institute of Hematology, West China Hospital, Sichuan University, #37 Guo Xue Xiang Street, Chengdu, 610041 China; 2 Sichuan University, Chengdu, China

**Keywords:** High risk, Maintenance, Myeloma, Network meta-analysis, Real-world study

## Abstract

Although the significant strides in novel therapeutic approaches have prolonged the survival of multiple myeloma (MM) patients, the unfavorable prognosis of cytogenetically high-risk newly diagnosed MM (NDMM) remains intractable with the lack of consensus regarding the choice of maintenance regimens. Therefore, this study was initiated with the aim of examining the effectiveness of various maintenance treatments for this group of patients in jeopardy. Overall, 17 studies with 1937 high-risk NDMM patients were included in the network meta-analysis. Combination therapies involving novel drugs presented encouraging prospects in the maintenance phase, while the patients and circumstances for the application of different regimens still needed to be further distinguished and clarified. To investigate the current status of maintenance therapy of high-risk NDMM patients in clinical practice, a real-world cohort of high-risk NDMM was retrospectively incorporated 80 patients with lenalidomide maintenance and 53 patients with bortezomib maintenance, presenting the median PFS of 31.7 months and 30.4 months, respectively (*p* = 0.874, HR = 0.966, 95% CI: 0.628–1.486). Collectively, this study illuminated the present constraints of conventional approaches during the maintenance phase for high-risk NDMM patients while highlighting the future potential associated with enhanced regimens integrating novel medications.

**Supplementary Information:**

The online version contains supplementary material available at 10.1007/s10238-024-01445-6.

## Introduction

Multiple myeloma (MM) manifests as the second most prevalent hematological malignancy that presents a pattern of ‘remission-relapse’ cycles [1], where relapses are inevitable with each subsequent remission of increasingly shorter duration, ultimately leading to the patient’s death due to the disease itself or treatment-related complications [2]. As a result, the plateau period after first-line treatment for newly diagnosed MM (NDMM) patients has garnered increasing attention. In order to extend the remission time and deepen the response to the maximum degree, maintenance treatment plays a crucial role in the overall treatment.

Across the preceding 15 years, the amelioration of survival outcomes among MM patients has been realized through the proliferation of therapeutic options, resulting in the observation of a median progression-free survival (mPFS) period ranging from 3 to 5 years, and a median overall survival (mOS) of 8 to 10 years [3]. However, roughly 20% of high-risk patients with an invasive disease course will die within 3 years of diagnosis [4]. Although the definition of high-risk MM is still under issue, the impact of high-risk cytogenetic abnormalities (HRCAs) on the prognosis of NDMM patients has been widely acknowledged, which encompasses del(17p), t(4;14), t(14;16), t(14;20), and gain(1q) [5]. Aggressive long-term maintenance therapy is regarded as an essential stage in order to overcome the substantially increased risk encountered by cytogenetically high-risk NDMM patients [6].

At present, maintenance therapy for NDMM has reached a stage where lenalidomide (Len) is established as the standard of care, with recommended maintenance regimens also containing bortezomib (Btz), daratumumab (Dara), ixazomib (Ixa), carfilzomib (Car) and dexamethasone (Dex) [7, 8]. However, as the historical use of interferon and glucocorticoids transitioned to the era of novel regimens, the maintenance strategy for patients with high-risk NDMM has become a debate. Thalidomide (Thal) has been stripped of recommendation attributed to its deleterious impact on overall survival (OS) in high-risk MM patients, as evidenced by the results of the MYELOMA IX trial in 2017 [9]. Recently, the utility of Btz monotherapy or dual regimens has been proposed as a more advisable option [10, 11]. Nevertheless, the superiority of Btz-based combination regimens over Btz or Len monotherapy has not been validated by prospective randomized trials [12, 13]. In the updated NCCN guideline for MM, dual maintenance was given additional emphasis for high-risk patients. Given the recent introduction of novel anti-MM agents with distinct mechanisms of action, the choice of maintenance therapy for high-risk NDMM patients becomes more equivocal for physicians against the backdrop of limited head-to-head comparisons. Despite the availability of some previous studies concerning maintenance therapies which incorporated outdated regimens like Thal and interferon and was exclusive of novel agents nowadays, evidence was obsolete and insufficient for current clinical decision-making [14]. Under such circumstances, the strategy for maintenance treatment for high-risk NDMM patients remains opaque. To assess the efficacy of different maintenance therapies for cytogenetically high-risk NDMM patients, a Bayesian network meta-analysis (NMA) was undertaken. Furthermore, a real-world cohort was incorporated to investigate the efficacy of commonly used maintenance regimens for high-risk NDMM patients in clinical practice.

## Method

### Network *meta*-analysis

The systematic review and NMA was reported according to the Preferred Reporting Items for Systematic reviews and Meta-Analyses (PRISMA) Statement and the extension statement for network meta-analysis (PRISMA-NMA) [15, 16]. Registration of the study was completed at the International Prospective Register of Systematic Reviews (PROSPERO, ID: CRD42022381459).

PubMed (MEDLINE), Embase (Ovid platform) and Web of Science were comprehensively searched for pertinent randomized controlled trials (RCTs). Conference abstracts were manually searched for gray literature. The search was confined to studies published in English prior to April 1, 2024. The detailed search strategy, criteria for study selection and procedure on data extraction are outlined in Supplemental Methods.

The evaluation of quality was conducted through meticulous utilization of the Cochrane Risk of Bias 2.0 tool (RoB2) [17]. Potential bias within individual studies was appraised by two independent reviewers (X.G. and W.T.) with any inconsistencies settled by a third researcher (L.Z.).

### Real-world study

A real-world, retrospective analysis on a cohort of cytogenetically high-risk NDMM patients was performed to reflect a more realistic maintenance pattern. Patients were enrolled from February 2018 to December 2022 at West China Hospital, and the final follow-up ended on May 1, 2024. The definition of the high cytogenetic risk group rested upon the Mayo Stratification of Myeloma and Risk-Adapted Therapy, with the existence of t (14;16), t (4;14), t (14;20), del (17p), p53 mutation and gain (1q) detected by fluorescent in situ hybridization [18]. Patients were classified by their initial maintenance therapy. Detailed study design is described in Supplemental Methods. The research adhered rigorously to the principles of the Declaration of Helsinki. The Ethics Review Committee of the Chinese Clinical Trial Registry granted approval for this study (No. ChiECRCT20190119). Informed consent was exempted by the ethics committee due to the nature of a retrospective study. Anonymization of all data was conducted before granting access to the authors.

### Statistical analysis

In the NMA, a Bayesian network framework was employed, utilizing a Monte Carlo Markov Chain (MCMC) model with 4 MCMC chains running simultaneously [19]. The trace plot and the density plot with bandwidth value were used to explore the appropriate adaptation and iteration number [20]. The Brooks–Gelman–Rubin diagnosis plot and potential scale reduction factor were used for qualitative and quantitative evaluation of the convergence degree of the ultimate model.

Risk ratio (RR) was calculated for comparisons of rates of dichotomous outcomes with a 95% credible interval (CrI), and hazard ratio (HR) was applied for analyzing survival outcomes based on time series. Deviation information criteria (DIC) were calculated to compare the differences between fixed-effect and random-effect frameworks. The overall heterogeneity of the model was assessed by the size of the heterogeneity variance parameter I^2^ based on the Q test [21], and the selection between fixed and randomized-effect models was based on consideration of both I^2^ and DIC differences. Network estimations were visualized by implementing league tables of the relative treatment effects. Post hoc subgroup analyses were conducted for autologous stem cell transplant (ASCT) status. Funnel plots were employed to examine the symmetry and potential asymmetry of data distribution [22]. The Bayesian NMA was conducted with package *Gemtc* in R software (version 4.1.2, The R Foundation).

In the real-world analysis, progression-free survival (PFS) entailed an assessment that spanned from the point of initial diagnosis to the occurrence of disease progression, mortality, or the last follow-up. Kaplan–Meier method was applied to describe survival, along with log-rank test for comparison between groups. HR accompanied by corresponding 95% confidence intervals (CI) was executed through the application of univariate Cox regression analysis. Statistical significance was ascertained with a two-sided *α* error of 0.05. Package *Survival* and *Survminer* in R software (version 4.1.2, The R Foundation) were utilized. The detailed statistical analysis plan is stated in Supplemental Methods.

## Results

### Results of literature search and quality assessments

**Fig. 1:**
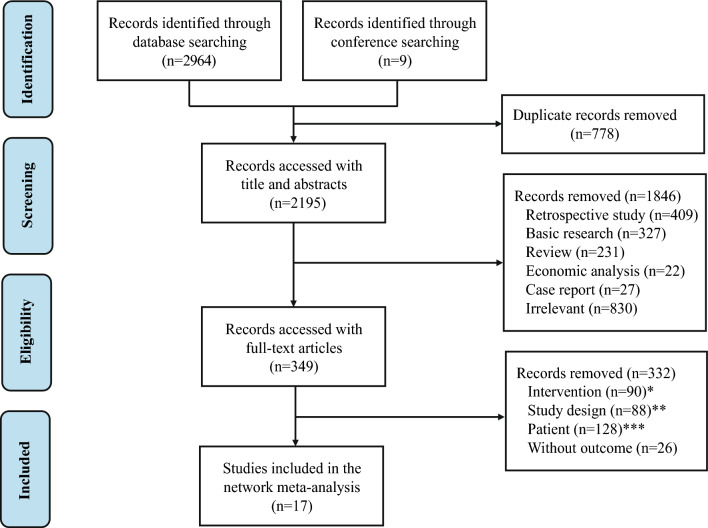
PRISMA flowchart of identification and selection process for eligible studies. PRISMA, Preferred Reporting Items for Systematic reviews and Meta-Analyses; NDMM, Newly diagnosed multiple myeloma. *Among the 90 excluded studies, 34 studies did not distinguish between different maintenance regimens, 31 studies did not reach the maintenance phase, and 25 studies incorporated thalidomide or interferon. **Among the 88 excluded studies, 27 studies were single-arm, and 61 studies only reported the comparison between high-risk and standard-risk NDMM under the same regimen, without results between high-risk patients under different maintenance regimens. ***Among the 128 excluded studies, 38 studies were specific to RRMM, 81 studies reported no results for high-risk patients, and 9 studies only presented results for NDMM patients with a certain kind of high-risk cytogenetic abnormality without analysis of all high-risk NDMM patients

After database searching, 2964 articles were retrieved, with an additional 9 manually added conference abstracts. Among them, 778 were duplicate records. Subsequently, 1846 articles were removed based on title and abstract, leaving 349 articles for the full-text appraisal (Fig. 1). Ultimately, 17 RCTs were deemed suitable for inclusion with the characteristics summarized (Table 1**, **Table S1) [23–39]. Of note, the studies were not included that did not perform head-to-head comparisons of different regimens, but only reported comparative results between standard-risk and high-risk patients receiving the same regimen. Additionally, real-world studies that presented head-to-head comparisons of different maintenance regimens were documented, and 3 studies were collected ultimately [40–42]. In an attempt to incorporate all trials within the same network, it was assumed that the relative efficacy of using either placebo or observation alone was consistent and included as the same intervention, denoted as Pbo/Obs. A total of 1937 cytogenetically high-risk NDMM patients and 11 different maintenance regimens were involved, including 3 triple-drug combinations, carfilzomib, lenalidomide plus dexamethasone (CarLenDex) and daratumumab, lenalidomide plus dexamethasone (DaraLenDex) and ixazomib, lenalidomide plus dexamethasone (IxaLenDex), 5 dual-drug combinations based on Len, carfilzomib plus lenalidomide (CarLen), daratumumab plus lenalidomide (DaraLen), ixazomib plus dexamethasone (IxaLen), lenalidomide plus prednisone (LenPdn), and lenalidomide plus prednisone (LenDex), as well as 3 distinct single-agent maintenance regimens, Len, Dara, and Ixa. The quality assessment of all studies was summarized (Fig. S1). Ten open-label studies aroused some concerns in terms of deviations from the intended interventions [24, 25, 28–30, 33–35, 38, 39].

**Table 1:** Trial details and patient characteristics of included studies

Study	Year	ID	Case (N.pts)	Control (N.pts)	Maintenance duration	Median follow-up time	ASCT	Phase	Age (median/range)	Definition of HRCA	Efficacy
IFM 2005	2012	NCT00430365	Len (52)	Pbo/Obs (29)	Until PD	55 months from diagnosis 45 months from consolidation	Yes	III	55 (22–67)	del(17p), t(4;14)	PFS
TOURMALINE-MM3	2018	NCT02181413	Ixa (61)	Pbo/Obs (54)	24 months or until PD	31 months from maintenance (IQR 27.3–35.7)	Yes	III	58 (52–64)	del(17p), t(4;14), t(14;16)	PFS
MYELOMA XI	2018	ISRCTN49407852	Len (166)	Pbo/Obs (113)	Until PD	31 months from maintenance (IQR 18–50)	Mixed	III	66 (59–72)	del(17p), t(4;14), t(14;16), t(14;20), gain(1q)	PFS, OS
FIRST	2018	NCT00689936	LenDex (43)	Pbo/Obs (47)	Until PD	67 months from induction (range 0–86.8)	No	III	73 (40–92)	t(4;14), t(14;16), del(17p)	PFS, OS
ALCYONE	2019	NCT02195479	Dara (53)	Pbo/Obs (45)	Until PD	40.1 months from induction (IQR 37.4–43.1)	No	III	71 (40–93)	del(17p), t(4;14), t(14;16)	PFS, OS
EMN01	2019	NCT01093196	LenPdn (37)	Len (36)	Until PD	71 months from induction	No	III	73 (50–89)	del(17p), t(4;14), t(14;16)	PFS, OS
HOVON-126	2020	NTR4910	Ixa (6)	Pbo/Obs (6)	Until PD	23.4 months from maintenance (range 6.9–35.5)	No	II	73 (66–82)	del(17p), t(4;14), t(14;16)	PFS
TOURMALINE-MM4	2020	NCT02312258	Ixa (150)	Pbo/Obs (91)	24 months or until PD	21.1 months from maintenance	No	III	72 (42–90)	del(17p), t(4;14), t(14;16), gain(1q)	PFS
CASSIOPEIA	2021	NCT02541383	Dara (57)	Pbo/Obs (70)	24 months or until PD	44.5 months from induction (IQR 38.9–49.1) 35.4 months from maintenance (IQR 30.2–39.9)	Yes	III	59 (53–63)	del(17p), t(4;14)	PFS
FORTE	2021	NCT02203643	CarLen (39)	Len (44)	24 months or until PD	50.9 months from induction (IQR 45.7–55.3) 37.3 months from maintenance (IQR 32.9–41.9)	Mixed	II	56 (51–62)	del(17p), t(4;14), t(14;16)	PFS
TOURMALINE-MM2	2021	NCT01850524	IxaLen (134)	Len (146)	Until PD	53.3 months from induction (IR group) 55.8 months from induction (R group)	No	III	74 (48–90)	t(4;14), t(14;16), del(17p), amp(1q)	PFS
RV-MM-PI-0752	2021	NCT02215980	LenDex (13)	Len (17)	Until PD	37 months (range 27–45 months)	No	III	76 (73–79)	t(4;14), t(14;16), del(17p)	PFS
MAIA	2021	NCT02252172	DaraLenDex (48)	LenDex (44)	Until PD	56.2 months from induction (IQR 52.7–59.9)	No	III	74 (70–78)	t(4;14), t(14;16), del(17p)	PFS, OS
GRIFFIN	2022	NCT02874742	DaraLen (42)	Len (37)	24 months or until PD	49.6 months	Yes	II	60 (29–70)	del(17p), t(4;14), t(14;16), t(14;20), gain/amp(1q)	PFS
ATLAS	2023	NCT02659293	CarLenDex (21)	Len (18)	Until PD	33·8 months (IQR 20·9–42·9)	Yes	III	59 (49–63)	del(13q), t(4;14), t(14;16), del(17p), hypodiploidy	PFS
GEM2014MAIN	2023	NCT02406144	IxaLenDex (31)	LenDex (33)	24 months for MRD- pts 50 months for MRD + pts	69 months from maintenance	Yes	III	58 (32–67)	t(4;14), t(14;16), del(17p)	PFS
PERSEUS	2023	NCT03710603	DaraLen (76)	Len (78)	Until PD	47.5 months from induction (range 0–54.4)	Yes	III	60 (31–70)	t(4;14), t(14;16), del(17p)	PFS

*N.pts* Number of patients, *ASCT*, autologous stem cell transplant, *HRCA* high-risk cytogenetic abnormality, *PD* progression disease, *PFS* progression-free survival, *OS* overall survival, *IQR* interquartile range, *CarLenDex* carfilzomib lenalidomide plus dexamethasone, *CarLen* carfilzomib plus lenalidomide, *DaraLenDex* daratumumab, lenalidomide plus dexamethasone, *DaraLen* daratumumab plus lenalidomide, *IxaLenDex* ixazomib lenalidomide plus dexamethasone, *IxaLen* ixazomib plus dexamethasone, *LenPdn* lenalidomide plus prednisone, *LenDex* lenalidomide plus dexamethasone, *Ixa* ixazomib; *Len*, lenalidomide, *Dara* daratumumab, *Pbo/Obs* placebo or observation

### Summarization of NMA results

**Fig. 2:**
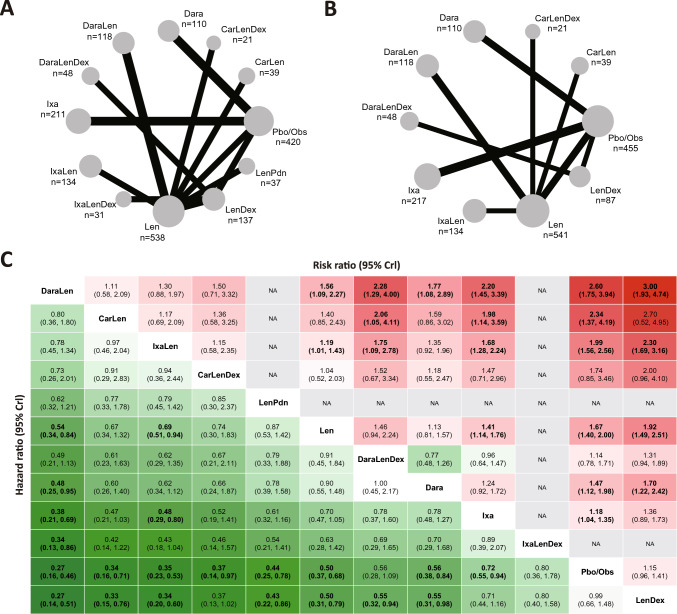
Results of PFS for high-risk NDMM patients who received maintenance therapy. **A** Network plot of HR in respect of PFS; **B** network plot of RR in respect of PFS; **C** league table presenting comparisons of the relative effects between each pair of interventions with both HR and RR results with corresponding 95% CrIs. HR, hazard ratio; RR, risk ratio; PFS, progression-free survival; NDMM, newly diagnosed multiple myeloma; CrI, credible interval

Network plots were used to display the direct comparisons regarding PFS among 12 treatments of HR analysis with 1844 patients (Fig. 2A) and 11 treatments of RR analysis with 1770 patients (Fig. 2B). Synthesized results were summarized as a league table (Fig. 2C). Given that 10 included studies were not blinded, the exact rankings could be misleading in real clinical practice. Nonetheless, considering the overall reference value of the analysis, regimens incorporating novel drugs will be interpreted in the order of the league table.

Two randomized, open-label trials examined both DaraLen maintenance and Len monotherapy with a total of 233 patients [29, 38]. In the PERSEUS study, DaraLen exhibited application potential in transplant-eligible, high-risk NDMM patients (HR = 0.38, 95% CI: 0.14–1.01). Furthermore, according to the results of NMA, DaraLen showed a significant reduction in the risk of relapse (HR = 0.54, 95% CrI: 0.34–0.84) than Len monotherapy. However, the GRIFFIN trial revealed that DaraLen could not entirely overcome the adverse impact of HRCAs (HR = 0.38, 95% CI: 0.14–1.01). Notably, in both trials, the DaraLen group received daratumumab, bortezomib, lenalidomide, and dexamethasone (Dara-VRD) in the induction phase, whereas the Len-maintenance group received Dara-free induction treatment. Therefore, the survival benefit should not be solely attributed to the use of Dara during the maintenance phase, but it was reinforced that continued use after induction could maximize the effect of Dara.

CarLen maintenance was evaluated in the FORTE trial, where high-risk NDMM patients received randomization before CarLen or Len maintenance [33]. CarLen did not yield superior PFS to Len standard maintenance across different HRCA groups: 0 HRCA (HR = 0.41, 95% CI: 0.16–1.04), 1 HRCA (HR = 0.70, 95% CI: 0.36–1.35), or 2 + HRCAs (HR = 0.53, 95% CI: 0.24–1.15). Synthesized results in the NMA also corroborated these findings (HR = 0.67, 95% CrI: 0.34–1.32). Typically, as the number of HRCA increases, PFS decreases in the maintenance phase. In the Len group, the 3-year PFS dropped from 67% for single-hit patients to only 42% for multiple-hit patients. However, it is worth noting that the 3-year PFS decreased from 69% to 67% in the CarLen group, which indicated the pronounced effect of Car-based maintenance in ultra-high-risk NDMM patients after exposure to proteasome inhibitors (PIs).

In the double-blind, placebo-controlled TOURMALINE-MM2 trial, IxaLen demonstrated superior efficacy over Len (HR = 0.69, 95%CI: 0.51–0.94) among transplant-ineligible, high-risk NDMM patients [32]. Results from NMA suggested a consistent trend. Nevertheless, considering that the IxaLen maintenance group and Len monotherapy group received Ixa-involved and Ixa-free induction regimens, respectively, and did not undergo secondary randomization before maintenance, this advantage could derive from a cumulative effect of successive use of Ixa.

According to the ATLAS trial, the triple maintenance regimen, CarLenDex, did not significantly reduce the risk of relapse compared with Len monotherapy (HR = 0.74, 95% CI: 0.30–1.86) among post-ASCT, high-risk NDMM patients [35]. However, the advantages of CarLenDex maintenance in the standard-risk group were unfolded (HR = 0.37, 95% CI: 0.20–0.70). Considering that patients who had received any kind of induction therapy could be included in the trial, more evidence was in need to validate the effect of triplet maintenance.

The MAIA study accessed the effect of successive use of DaraLenDex versus LenDex in transplant-ineligible, high-risk NDMM patients (HR = 0.55, 95%CI: 0.32–0.94) [39]. It is worth noticing that all the patients were over 65 years old, indicating that Dara-based combination regimens could be promising options for this poorly tolerated cohort with limited treatment modalities.

In terms of IxaLenDex, no evidence was found that the addition of Ixa would lead to a better prognosis than LenDex (HR = 1.26, 95% CI: 0.64–2.50) in the GEM2014MAIN study [37]. The 6-year PFS for high-risk NDMM patients receiving IxaLenDex and LenDex maintenance was 38.7% and 50.2%, respectively. Nevertheless, in the TOURMALINE-MM2 study, 40.3% of IxaLen-maintained patients and 28.8% of Len-maintained patients did not experience relapse [32]. On one hand, not undergoing maintenance therapy is highly disadvantageous. On the other hand, the concurrent use of corticosteroids might not necessarily confer the anticipated benefits to high-risk patients.

Of note, no evidence of improved OS was found through analysis of both HR (Fig.S2) and RR (Fig. S3). According to post hoc subgroup analyses, no evidence was found that different ASCT statuses would affect the selection of maintenance regimens (Figs. S4–S7).

### Outcomes of real-world analysis

**Fig. 3:**
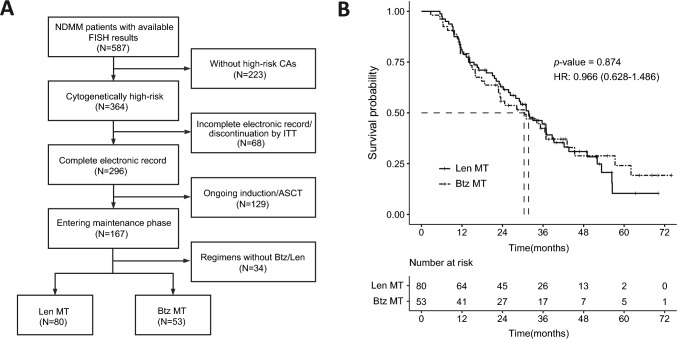
Real-world analysis of high-risk NDMM patients treated with Len-based or Btz-based maintenance therapies. **A** Patient selection flow chart; **B** PFS by maintenance regimens. FISH, fluorescent in situ hybridization; CA, cytogenetic abnormality; ITT, intention to treat; ASCT, autologous stem cell transplant; Len, lenalidomide; Btz, bortezomib; MT, maintenance; HR, hazard ratio; PFS, progression-free survival

The patient selection process was summarized in the real-world analysis (Fig. 3A). Finally, a total of 133 patients diagnosed as high-risk NDMM were encompassed within the scope of the real-world analysis, of which 80 patients received Len maintenance (10 mg Len once daily on days 1–21 of a 28-day cycle) and 53 patients received Btz maintenance (1.3 mg/m^2^ subcutaneous Btz every 2 weeks). The median duration of follow-up spanned 26.3 months (interquartile range 14.0–38.9 months). The median PFS for Len-based and Btz-based maintenance was 31.7 months and 30.4 months, respectively (Fig. 3B**)**, and the disparity did not achieve statistical significance (*p* = 0.874, HR = 0.966, 95% CI: 0.628–1.486).

## Discussion

This study underlined the predicament that current standard maintenance therapy was not effective in overcoming relapse in cytogenetically high-risk NDMM patients. However, in the context of the era of new drugs, combination regimens containing novel drugs showed encouraging application prospects in the maintenance phase.

Notably, the maintenance regimen based on Len has great potential in treating single-hit, high-risk NDMM, although the negative impact of multiple hits cannot be overcome entirely. In the DETERMINATION trial, patients with high-risk NDMM who received Len maintenance still tolerated a higher risk of progression than standard-risk patients (HR = 1.55, 95% CI: 1.17–2.05) [43]. However, the 24-month PFS rates for standard-risk, 1 HRCA and ≥ 2 HRCAs patients in the MASTER trial who received Len maintenance treatment were 92%, 96%, and 66%, respectively [44]. In the GRIFFIN trial, the 48-month PFS rates were 94% for standard-risk, 91% for 1 HRCAs, and 54% for ≥ 2 HRCAs subgroups who received DaraLen as maintenance therapy [45]. Selecting Len-based maintenance regimens according to different numbers of HRCA could be a viable solution [46]. More evidence is needed to establish Len as the backbone drug for maintenance treatment in high-risk subgroups.

While Btz was another maintenance option commonly employed for high-risk NDMM patients, the number of relevant clinical trials was scarce. The only few studies involving Btz were not included in the analysis because they did not meet the inclusion criteria. The HOVON-65/GMMG HD4 study indicated that whereas Btz maintenance may benefit high-risk NDMM patients, particularly in the context of del(17p), its efficacy to prolong PFS and OS of patients with other HRCAs was limited, such as t(4;14) and del(13q) [47]. In a retrospective study, the mPFS of high-risk NDMM patients were 27 months for Len and 28 months for Btz maintenance [42]. Additionally, a real-world comparison of patients in both the GMMG-HD4 and MM5 trial discovered no significant difference between the impact of Len and Btz maintenance on PFS and OS of high-risk patients [40]. More recently, the Len and Btz monotherapy yielded the mPFS of 26.9 and 20.0 months among the real-world NDMM patients with HRCAs, respectively [48]. The non-significant difference in efficacy between Len and Btz in the real world was further validated by more recent study that included 196 high-risk NDMM patients [41].

The effect of Btz-based combination regimens in high-risk NDMM patients also encountered controversy. In the GEM2005MAS65 trial, the efficacy of two Btz-based maintenance regimens in high-risk patients was investigated, bortezomib plus thalidomide (BtzThal) and bortezomib plus prednisone (BtzPdn) [49]. Both regimens yielded comparable prognosis, with mPFS of 28 months for BtzThal and 27 months for BtzPdn, and CR rates of 48% and 41%, respectively. Moreover, a phase III clinical trial from GIMEMA demonstrated that the BtzThal produced comparable results to no maintenance in high-risk patients [50]. A CIBMRT study revealed that Btz plus Len did not produce superior clinical benefits than Len maintenance in high-risk post-ASCT NDMM patients, especially those with abnormalities at chromosome 1q [51]. The limitations of Btz-based maintenance regimens still exist, suggesting the urgency of addressing the therapeutic challenges faced by high-risk NDMM patients.

It is worth noting that some new drugs have gradually shown their edges for high-risk patients in the realm of maintenance therapy. The NMA conducted by the Mayo Clinic in post-ASCT NDMM patients uncovered that CarLenDex and CarLen significantly prolonged PFS in comparison with Len, Thal, Ixa and Dara [52]. Another NMA based on ASCT-ineligible NDMM patients suggested that daratumumab, lenalidomide plus dexamethasone (DaraLenDex) engendered a substantial improvement in PFS and OS compared to the LenDex-continuous regimen and emerged as the most effective treatment among all [53]. According to the GEM2012MENOS65 trial, receiving IxaLen as maintenance therapy after achieving undetectable minimal residual disease through next-generation flow cytometry could potentially mitigate the poor prognosis associated with multiple adverse factors, including HRCAs [54]. Additionally, the role of Car-based maintenance in deepening responses and prolonging PFS was supported by more studies [55, 56]. Currently, the MajesTEC-4 (EMN30) study which compares teclistamab in combination with Len versus Len monotherapy has advanced to the maintenance phase without available outcomes [57]. Considering the indefinite endurance, the choice of drugs for maintenance therapy ought to be evaluated thoroughly in terms of economics, side effects, long-term prognosis, and impact on quality of life. Therefore, further researches that consider extended evaluation criteria are essential to outline a clear roadmap for effective and safe maintenance regimens.

To our knowledge, this study included the first NMA that involves all types of high-risk NDMM patients, including both post-ASCT and ASCT-ineligible cohorts. This study is also the most comprehensive analysis of the maintenance regimens to date and provides the most extensive landscape of both the clinical practice and the investigational phase. Besides, subgroup analyses were performed to further explore differences in the efficacy of maintenance regimens in high-risk patients with different ASCT statuses. In addition, real-world analysis complemented and demonstrated the insufficiency of existing maintenance regimens in clinical practice.

Some limitations should be considered when generalizing the conclusions of this study. Firstly, the absence of a universally recognized definition of HRCAs restricted this analysis to the reliance on the descriptions provided in included studies, which may introduce a certain degree of heterogeneity. Secondly, 8 studies in the NMA did not undergo further randomization after prior treatment, which could have influenced the comparison of maintenance therapy due to the potential impact of induction and/or consolidation regimens [23, 24, 26, 29, 30, 32, 36, 39]. Only the ALCYONE study [30] detailed the pre-maintenance state of patients with HRCAs, and the CASSIOPEIA [34], TOURMALINE-MM3 [26], and MM4 [27] study solely included patients with a partial or greater response. Moreover, as there are only a limited number of head-to-head clinical trials reporting maintenance outcomes for high-risk subgroups, the NMA focuses on the impact of different regimens of maintenance on high-risk NDMM, without further elaborating the different usage and dose of drugs. In the CASSIOPEIA trial [34], Dara was given as a 16 mg/kg intravenous injection once every eight weeks, while the same dose was administered by a four-week cycle in the ALCYONE study [30]. A subcutaneous injection of 1800 mg once every four weeks was utilized in the PERSEUS study [38]. The GRIFFIN study includes all three dosages above [29]. This heterogeneity in the administration of drugs posed a challenge in further evaluating the impact of different dosing schedules on the effectiveness of maintenance therapy. Finally, the single-center, real-world part had a limited sample size, but the results corroborated with three previous studies with an overall 362 high-risk patients [40–42]. Above all, this study sheds insights into the appropriate selection of maintenance regimens for high-risk NDMM patients, offering a comprehensive list of the drugs with the potential to attenuate or even counteract the serious repercussions of HRCAs.

## Conclusions

In brief, standard monotherapy for maintenance was not adequate to sufficiently reduce relapse risk in cytogenetically high-risk NDMM patients. Importantly, combination regimens incorporating novel drugs demonstrated high potential to prolong PFS, warranting further exploration of doublet and triplet maintenance for this group of patients with unfavorable prognosis. Moreover, the incorporation of corticosteroids into maintenance regimens did not yield anticipated advantages, which may be associated with adverse events from continuous use, which necessitated further investigation to provide additional evidence.

## Supplementary Information

Below is the link to the electronic supplementary material.

Supplementary file1 (PDF 3840 kb)

## Data Availability

The data set used and/or analyzed during the current study is available from the corresponding author on reasonable request.
