# Supplementary material for: Maintenance therapy for cytogenetically high-risk multiple myeloma: landscape in the era of novel drugs
Source: Clin Exp Med. 2024 Aug 6;24(1):179. doi: 10.1007/s10238-024-01445-6 (PMC11303491; doi:10.1007/s10238-024-01445-6)
Supplement: Supplementary file 1 — Supplementary file1 (PDF 3840 kb) [file 10238_2024_1445_MOESM1_ESM.pdf]

Supplemental Materials

Maintenance therapy for cytogenetically high-risk multiple myeloma: landscape in the era of novel drugs

Table of contents

Appendix 1: Supplemental Methods **2**

Part I: Network meta-analysis

Part II: Real-world analysis

Appendix 2: Quality Assessment **8**

Supplemental Figure 1

Supplemental Table 1

Supplemental Table 2

Supplemental Table 3

Supplemental Figure 2-7

Appendix 4: Heterogeneity Test and Funnel Plot **19**

Supplemental Figure 8-10

Appendix 5: Rationale for the RoB 2 Assessment **22**

References **38**

Appendix 1: Supplemental Methods

Part I: Network meta-analysis

Literature search strategy

PubMed (MEDLINE), Embase (Ovid platform) and Web of Science were comprehensively searched for pertinent randomized controlled trials (RCTs). Conference abstracts from the American Society of Hematology (ASH), the European Hematology Association (EHA), and the American Society of Clinical Oncology (ASCO) were manually searched for grey literature. The search was limited to studies published in English prior to 2024.4.1. The keywords were “multiple myeloma”, “newly diagnosed” and “maintenance”. Notably, for the results of cytogenetically high-risk newly diagnosed multiple myeloma (NDMM) patients are usually reported in the body section or supplemental material as a subgroup analysis, "high-risk" was not applied as a keyword in the literature search.

Database	Search Number	Query	Results
Pubmed	1	((((((((((((((((((((Multiple Myeloma*[Title/Abstract]) OR Multiple Myelomas*[Title/Abstract]) OR Myelomas, Multiple*[Title/Abstract]) OR Myeloma, Multiple*[Title/Abstract]) OR Myeloma, Plasma-Cell*[Title/Abstract]) OR Myeloma, Plasma Cell*[Title/Abstract]) OR Myelomas, Plasma-Cell*[Title/Abstract]) OR Plasma-Cell Myeloma*[Title/Abstract]) OR Plasma-Cell Myelomas*[Title/Abstract]) OR Myelomatosis*[Title/Abstract]) OR Myelomatoses*[Title/Abstract]) OR Plasma	51,685

		Cell Myeloma*[Title/Abstract]) OR Cell Myeloma, Plasma*[Title/Abstract]) OR Cell Myelomas, Plasma*[Title/Abstract]) OR Myelomas, Plasma Cell*[Title/Abstract]) OR Plasma Cell Myelomas*[Title/Abstract]) OR Kahler Disease*[Title/Abstract]) OR Disease, Kahler*[Title/Abstract]) OR Myeloma-Multiple*[Title/Abstract]) OR Myeloma Multiple*[Title/Abstract]	
	2	((Newly Diagnosed* [Title/Abstract]) OR Newly-diagnosed* [Title/Abstract]) OR NDMM [Title/Abstract]	66,320
	3	(((Maintenance* [Title/Abstract]) OR Maintain* [Title/Abstract]) OR Continuous* [Title/Abstract]) OR Continue* [Title/Abstract]	2,118,261
	4	#1 AND #2 AND #3	688
Embase	1	('multiple myeloma*' OR 'kahler disease*' OR 'morbus kahler*' OR 'myeloma multiplex*' OR 'myelomatosis*' OR 'plasma cell myeloma*').ti.	53,014
	2	('newly diagnosed*' OR 'newly-diagnosed*' OR 'NDMM*').ti.	24,827
	3	('maintenance*' OR 'maintain*' OR 'continuous*' OR 'continue*').ti.	170,618
	4	#1 AND #2 AND #3	307
Web of Science	1	(((((TS=(multiple myeloma*)) OR TS=(kahler disease*)) OR TS=(morbus Kahler*)) OR TS=(myeloma multiplex*)) OR TS=(myelomatosis*)) OR TS=(plasma cell myeloma*)	145,507

	2	((TS=(newly diagnosed*)) OR TS=(newly-diagnosed*)) OR TS=(NDMM*)	107,758
	3	(((TS=(maintenance*)) OR TS=(maintain*)) OR TS=(continuous*)) OR TS=(maintain*)	10,524,359
	4	#1 AND #2 AND #3 and Clinical Trial (Document Types)	1,969

Study selection

Studies were eligible for inclusion in the network meta-analysis if they met all the following criteria: (1) study design: RCTs; (2) patients: confirmed with NDMM according to International Myeloma Working Group (IMWG) and with any high-risk cytogenetic abnormalities (CAs), including del(17p), t(4;14), t(14;16), t(14;20) and gain(1q); (3) intervention: any current maintenance therapies; (4) control: placebo, observation only, or any current maintenance therapies; (5) outcomes: survival endpoints of high-risk NDMM patients. Trials were excluded for three reasons: (1) studies published in languages other than English; (2) studies without a follow-up period of at least 12 months; (3) literature types including basic research, retrospective cohort study, case report, economic analysis or review; (4) studies incorporating obsolete maintenance regimens that have been eliminated by guidelines (Thal, interferon and glucocorticoids monotherapy). For studies that have published two or more papers, only the most recent or complete articles were examined. Two (X.G. and W.T.) independent investigators conducted a thorough screening of all the included studies using EndNote X9 (Clarivate Analytics, Philadelphia, PA, USA), and any discrepancies or disagreements were resolved by negotiation.

Data extraction and quality assessment

Two (X.G. and W.T.) independent reviewers extracted information from all the eligible studies with Microsoft Excel (Microsoft, Redmond, WA, USA): (1) basic

information of the study: study name, first author, year of publication, registration number, and study phase; (2) data related to patients and treatments: intervention (induction, consolidation and maintenance), dosage, duration of maintenance, duration of follow-up period, number of patients included, definition of cytogenetic high-risk, status of autologous stem cell transplant (ASCT) and the age of included patients; (3) survival outcomes: the number of events, hazard ratio (HR) and corresponding 95% confidence interval for both progression-free survival (PFS) and OS. The primary outcome was PFS, with OS serving as a secondary endpoint. All data were officially and directly reported in the literature, without any secondary estimation or calculation.

Statistical analysis

A Bayesian network framework was employed, utilizing a Monte Carlo Markov Chain (MCMC) model with 4 MCMC chains running simultaneously.[1] The trace plot and the density plot with Bandwidth value were used to explore the appropriate adaptation and iteration number.[2] The eventual number of adaptations was set up to 5000, and the number of iterations was set up to 20,000. The Brooks-Gelman-Rubin diagnosis plot and potential scale reduction factor (PSRF) were used for qualitative and quantitative evaluation of the convergence degree of the ultimate model.

Risk ratio (RR) was calculated for comparisons for rates of dichotomous outcomes with a 95% credible interval (CrI), and hazard ratio (HR) was applied for analyzing survival outcomes based on time series. Deviation information criteria (DIC) were calculated to compare the differences between fixed-effect and random-effect frameworks. If the difference of DIC was less than 5 between fixed-effect and random-effect frameworks, the two frameworks were considered to produce comparable results. The consistency analysis of direct and indirect comparisons would be conducted by the method of node splitting test if a closed-loop relationship exists in the network. The overall heterogeneity of the model was assessed by the size of the heterogeneity variance parameter I^2 based on Q test,[3] and the selection between fixed and randomized-effect models was based on consideration of both I^2 and DIC differences. For all

outcomes, we summarized the evidence by a network relation graph. To visualize comparisons of network estimations, league tables of the relative treatment effects were implemented. Post hoc subgroup analyses were conducted for ASCT status. Funnel plots were used to examine the potential publication bias of included studies.[4] This Bayesian NMA was conducted with package *Gemtc* in R software (version 4.1.2, The R Foundation).

Part II: Real-world analysis

Patients were enrolled from February 2018 to December 2022 at West China Hospital if they were newly diagnosed with symptomatic MM with high-risk CAs. The final follow-up ended in May 1, 2024. The study was conducted in accordance with the principles of the Declaration of Helsinki. Approval for this study was obtained from the Ethics Review Committee of the Chinese Clinical Trial Registry (No. ChiECRCT20190119). Informed consent was waived by the ethics committee due to a retrospective study. All of the data were anonymized prior to access by authors.

Patients were included if they meet the following criteria: (1) newly-diagnosed symptomatic MM confirmed according to IMWG; (2) high-risk CAs detected by fluorescent in situ hybridization (FISH); (3) NDMM patients received at least one cycle of induction treatment. The exclusion criteria were as follows: (1) age<18 years; (2) the presence of extramedullary disease or plasma cell leukemia; (4) patients with second primary carcinoma; (5) patients enrolled in a clinical trial; (6) incomplete demographic or clinical data; (7) maintenance regimens without bortezomib (Btz) or lenalidomide (Len). In this study, pre-determined patient information was collected. The demographic factors of age and gender were included, alongside relevant clinical information such as induction therapy, ASCT status, maintenance regimens, best response and risk groups. In addition, laboratory outcomes were also collected, including lactic dehydrogenase, hemoglobin, platelet, albumin and β2-macroglobulin at initial diagnosis.

The definition of the high cytogenetic risk group was based on the Mayo Stratification of Myeloma and Risk-Adapted Therapy,[5] with the presence of t(14;16), t(4;14), t(14;20), del(17p), p53 mutation and gain(1q) detected by FISH of the CD138-enriched plasma cells from the bone marrow. Double hit and triple hit were determined on the basis of the number of high-risk CAs detected. Different risk groups were classified using Durie and Salmon staging system (DS),[6] International Staging System (ISS),[7] and Revised International Staging System (R-ISS).[8] All patients were grouped according to different maintenance regimens: (1) Btz-based maintenance; (2) Len-based maintenance. Treatment schedule was as follows: Btz was administered 1.3 mg/m^2 subcutaneously every 2 weeks, Len was given 10 mg once daily on days 1–21 of a 28-day cycle. Both maintenance treatment was continued until disease progression, unacceptable toxicity or adjustment by ITT.

Progression-free survival (PFS) was measured from diagnosis to disease progression, death, or the last follow-up. Continuous data were presented as mean $\pm$ standard deviation (SD) or as the median and interquartile range (IQR) and compared with student t-test. Categorical variables were presented as the number of cases with frequency and compared using Chi-square or Fisher-test. For survival analysis, Kaplan-Meier method was applied for description and the log-rank test for comparison between groups. HR with 95% confidence intervals (CI) were calculated using univariate Cox regression analysis. Statistical significance was established with a two-sided α error of 0.05. Package *Survival* and *Survminer* in R software (version 4.1.2, The R Foundation) were utilized for survival analysis.

Appendix 2: Quality Assessment

Study ID	D1	D2	D3	D4	D5	Overall
IFM 2005	+	+	!	+	+	!
TOURMALINE-MM3	!	+	+	+	+	!
MYELOMA XI	+	!	+	+	+	!
FIRST	!	!	+	+	+	!
ALCYONE	+	!	+	+	+	!
EMN01	+	!	+	+	+	!
HOVON-126	!	+	+	+	+	!
TOURMALINE-MM4	+	+	+	+	+	+
CASSIOPEIA	+	!	+	+	+	!
FORTE	+	!	+	!	!	!
TOURMALINE-MM2	+	+	!	+	+	!
GRIFFIN	+	!	+	+	+	!
ATLAS	+	!	+	+	!	!
MAIA	+	!	+	+	+	!
RV-MM-PI-0752	!	+	+	+	!	!
GEM2014MAIN	!	!	+	+	+	!
PERSEUS	!	!	+	+	+	!

+ Low risk
! Some concerns
- High risk

D1 Randomisation process
D2 Deviations from the intended interventions
D3 Missing outcome data
D4 Measurement of the outcome
D5 Selection of the reported result

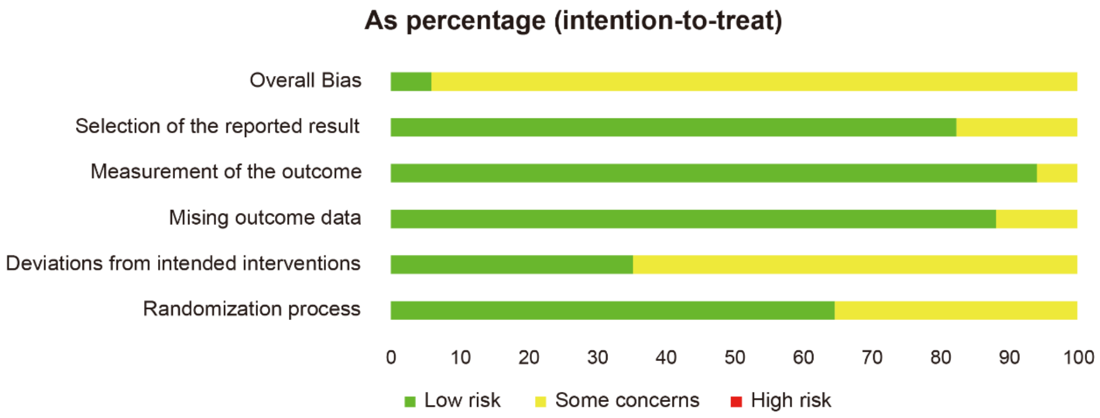

Supplemental Figure 1. Risk of bias of the included studies

Appendix 3: Supplemental Results

Supplemental Table 1. Detailed trial characteristics of included studies

Study	First author	Year	ID	Case (N.pts)	Control (N.pts)	Dosage	Maintenance duration	Median follow-up time	Induction	ASCT	Consolidation	Phase	N.random	Age (median/range)	Definition of HRCA	Efficacy
IFM 2005[9]	Attal	2012	NCT00430365	Len (52)	Pbo/Obs (29)	Len (10mg po. qd, increased to 15mg if tolerated)	Until PD	55 months from diagnosis<br>45 months from consolidation	Mixed (BtzCtxLenDoxDex, BtzDex, other)	Yes	2 cycles of Len	III	1	55 (22-67)	del(17p), t(4;14)	PFS
TOURMALINE-MM3[10]	Dimopoulous	2018	NCT02181413	Ixa (61)	Pbo/Obs (54)	Ixa (3mg po. d1,8,15; 4mg Cycles 5+ if tolerated)	24 months or until PD	31 months from maintenance (IQR 27.3-35.7)	Mixed	Yes	ND	III	1	58 (52-64)	del(17p), t(4;14), t(14;16)	PFS
MYELOMA XI[11]	Jackson	2018	ISRCTN49407852	Len (166)	Pbo/Obs (113)	Len (10mg po. d1-21)	Until PD	31 months from maintenance (IQR 18-50)	Mixed (CtxThalDex, CtxLenDex, CarCtxLenDex)	Mixed	2-8 cycles of BtzCtxDex or ND	III	3	66 (59-72)	del(17p), t(4;14), t(14;16), t(14;20), gain(1q)	PFS, OS
FIRST[12]	Facon	2018	NCT00689936	LenDex (43)	Pbo/Obs (47)	Len (2.5mg, 5mg, 10mg, 15mg, 20mg, or 25 mg po. d1-21)<br>Dex (40mg po. d1,8,15,22)	Until PD	67 months from induction (range 0-86.8)	LenDex group: 18 cycles of LenDex<br>Pbo/Obs group: 12 cycles of MelPdnThal	No	ND	III	1	73 (40-92)	t(4;14), t(14;16), del(17p)	PFS, OS
ALCYONE[13]	Mateos	2019	NCT02195479	Dara (53)	Pbo/Obs (45)	Dara (16mg/kg iv. q4w.)	Until PD	40.1months from induction (IQR 37.4-43.1)	Dara group: 9 cycles of DaraBtzMelPdn<br>Obs group: 9 cycles of BtzMelPdn	No	ND	III	1	71 (40-93)	del(17p), t(4;14), t(14;16)	PFS, OS
EMN01[14]	Bringhen	2019	NCT01093196	LenPdn (37)	Len (36)	Len (10mg po. d1-21)<br>Pdn (25mg po. qd.)	Until PD	71 months from induction	Mixed (LenDex, MelPdnLen, CtxPdnLen)	No	ND	III	2	73 (50-89)	del(17p), t(4;14), t(14;16)	PFS, OS
HOVON-126[15]	Zweegman	2020	NTR4910	Ixa (6)	Pbo/Obs (6)	Ixa (2-4mg po. d1,8,15)	Until PD	23.4 months from maintenance (range 6.9-35.5)	9 cycles of IxaThalDex	No	ND	II	1	73 (66-82)	del(17p), t(4;14), t(14;16)	PFS
TOURMALINE-MM4[16]	Dimopoulous	2020	NCT02312258	Ixa (150)	Pbo/Obs (91)	Ixa (3mg po. d1,8,15; 4mg Cycles 5+ if tolerated)	24 months or until PD	21.1 months from maintenance	Mixed (BtzCtxDex, BtzThalDex, LenDex, etc.)	No	ND	III	1	72 (42-90)	del(17p), t(4;14), t(14;16), gain(1q)	PFS
CASSIOPEIA[17]	Moreau	2021	NCT02541383	Dara (57)	Pbo/Obs (70)	Dara (16mg/kg iv. q8w.)	24 months or until PD	44.5 months from induction (IQR 38.9-49.1)<br>35.4 months from maintenance (IQR 30.2-39.9)	4 cycles of Dara-BtzThalDex or BtzThalDex	Yes	2 cycles of DaraBtzThalDex or BtxThalDex	III	2	59 (53-63)	del(17p), t(4;14)	PFS
FORTE[18]	Mina	2021	NCT02203643	CarLen (39)	Len (44)	Car (36mg/m^2 iv. d1-2,15-16)<br>Len (10mg po. d1-21)	24 months or until PD	50.9 months from induction (IQR 45.7-55.3)<br>37.3 months from maintenance (IQR 32.9-41.9)	4 cycles of CarLenDex or CarCtxDex	Mixed	4 cycles of CarLenDex or CarCtxDex	II	2	56 (51-62)	del(17p), t(4;14), t(14;16)	PFS
TOURMALINE-MM2[19]	Facon	2021	NCT01850524	IxaLen (134)	Len (146)	Ixa (3mg po. d1,8,15)<br>Len (10mg po. d1-21)	Until PD	53.3 months from induction (IxaLen group)<br>55.8 months from induction (Len group)	IxaLen group: 18 cycles of IxaLenDex<br>Len group: 18 cycles of LenDex	No	ND	III	1	74 (48-90)	t(4;14), t(14;16), del(17p), amp(1q)	PFS
RV-MM-PI-0752[20]	Larocca	2021	NCT02215980	LenDex (13)	Len (17)	Len (10mg po, d1-21)<br>Dex (20mg po. d1, 8, 5, 22)	Until PD	37 months (range 27-45 months)	Len (25mg po, d1-21)<br>Dex (20mg po. d1, 8, 5, 22)	No	ND	III	1	76 (73-79)	t(4;14), t(14;16), del(17p)	PFS
MAIA[21]	Facon	2021	NCT02252172	DaraLenDex (48)	LenDex (44)	Dara (16mg/kg iv. q4w Cycle 7+)<br>Len (25mg po. d1-21)<br>Dex (40mg po. d1, 8, 15, 22)	Until PD	56.2 months from induction (IQR 52.7-59.9)	DaraLenDex group: DaraLenDex<br>LenDex group: LenDex	No	ND	III	1	74 (70-78)	t(4;14), t(14;16), del(17p)	PFS, OS
GRIFFIN[22]	Callander	2022	NCT02874742	DaraLen (42)	Len (37)	Dara (14mg/kg iv. q8w. or q4w., or 1800mg sc. q4w. per protocol amendments)<br>Len (10mg po. d1-21; 15mg Cycles 10+ if tolerated)	24 months or until PD	49.6 months	Dara-Len group: 4 cycles of DaraLenBtzDex<br>Len group: 4 cycles of LenBtzDex	Yes	2 cycles of DaraLenBtzDex (DaraLen group)<br>2 cycles of LenBtzDex (Len group)	II	1	60 (29-70)	del(17p), t(4;14), t(14;16), t(14;20), gain/amp(1q)	PFS
ATLAS[23]	Dytfeld	2023	NCT02659293	CarLenDex (21)	Len (18)	Car (36mg/m^2 iv. d 1-2, 8-9,15-16)<br>Len (25mg po. d1-21)<br>Dex (20mg po. d1, 8, 15, 22)	Until PD	33·8 months (IQR 20·9–42·9)	Any type of induction	Yes	Any type of consolidation or ND	III	1	59 (49-63)	del(13q), t(4;14), t(14;16), del(17p), hypodiploidy	PFS
GEM2014MAIN[24]	Rosinol	2023	NCT02406144	LenDex (33)	IxaLenDex (31)	Ixa (4 mg po. d1, 8, and 15<br>Len (15 mg po. d1-21)<br>Dex (20 mg po. d1-4 and d9-12)	24/50 months for MRD-/+ pts	69 months from maintenance	6 cycles of BtzLenDex	Yes	2 cycles of BtzLenDex	III	1	58 (32-67)	t(4;14), t(14;16), del(17p)	PFS
PERSEUS[25]	Sonneveld	2023	NCT03710603	DaraLen (76)	Len (78)	Dara (1800mg sc. q4w)<br>Len (10mg po. qd)	Until PD	47.5 months from induction (range 0-54.4)	DaraLen group: 4 cycles of DaraLenBtzDex<br>Len group: 4 cycles of LenBtzDex	Yes	DaraLen group: 4 cycles of DaraLenBtzDex<br>Len group: 4 cycles of LenBtzDex	III	1	60 (31-70)	t(4;14), t(14;16), del(17p)	PFS

Abbreviations: *N.pts* Number of patients, *ASCT* Autologous stem cell transplant, *N.random* Number of randomization, *HRCA* High-risk cytogenetic abnormality, *PD* Progression disease, *PFS* Progression-free survival; *OS* Overall survival, *IQR* Interquartile range, *Car* Carfilzomib, *Pdn* Prednisone, *Dex* Dexamethasone, *Ixa* Ixazomib, *Len* Lenalidomide, *Dara* Daratumumab, *Btz* Bortezomib, *Ctx* Cyclophosphamide, *Thal* Thalidomide, *Dox* Doxorubicin, *Mel* Melphalan, *Pbo/Obs* Placebo or observation.

Supplemental Table 2. Summary of Bayesian network framework applied in the meta-analysis

Variant	Patients	Outcome	Model	DIC	I^2	Apply	PSRF
RR	All	PFS	Fixed	51.78850	9%	Yes	1
			Random	52.17597	3%	No	NA
		OS	Fixed	15.965765	14%	Yes	1
			Random	15.991670	14%	No	NA
	After ASCT	PFS	Fixed	21.92546	4%	Yes	1
			Random	22.62556	1%	No	NA
	Without ASCT	PFS	Fixed	18.698507	14%	Yes	1
			Random	18.197064	8%	No	NA
HR	All	PFS	Fixed	29.98087	26%	Yes	1
			Random	29.21886	10%	No	NA
		OS	Fixed	9.903830	19%	Yes	1
			Random	9.855086	19%	No	NA
	After ASCT	PFS	Fixed	4.595457	23%	Yes	1
			Random	5.143029	25%	No	NA
	Without ASCT	PFS	Fixed	13.992347	14%	Yes	1
			Random	13.996811	14%	No	NA

Abbreviations: *RR* Risk ratio, *DIC* Deviation information criteria, *PSRF* Potential scale reduction factor, *PFS* Progression-free survival; *OS* Overall survival, *ASCT* Autologous stem cell transplant, *HRCA* High-risk cytogenetic abnormality, *NA* Not applicable.

Supplemental Table 3. Summary of outcome data extracted from included studies

Study	Total		PFS			OS		
	Case (N.pts)	Control (N.pts)	Case (N.events)	Control (N.events)	HR (95% CI)	Case (N.events)	Control (N.events)	HR (95% CI)
IFM 2005[9]	Len (52)	Pbo/Obs (29)	Len (21)	Pbo/Obs (17)	NA	NA	NA	NA
TOURMALINE-MM3[10]	Len (61)	Pbo/Obs (54)	Len (38)	Pbo/Obs (38)	0.62 (0.38-1.02)	NA	NA	NA
MYELOMA XI[11]	Len (166)	Pbo/Obs (113)	Len (75)	Pbo/Obs (88)	0.45 (0.33-0.62)	Len (43)	Pbo/Obs (41)	0.89 (0.57-1.37)
FIRST[12]	LenDex (43)	Pbo/Obs (47)	LenDex (39)	Pbo/Obs (37)	1.27 (0.81–2.01)	LenDex (43)	Pbo/Obs (47)	0.92 (0.58, 1.46)
ALCYONE[13]	Dara (53)	Pbo/Obs (45)	Dara (24)	Pbo/Obs (19)	0.78 (0.43-1.43)	Dara (23)	Pbo/Obs (21)	0.91 (0.50-1.65)
EMN01[14]	LenPdn (37)	Len (36)	NA	NA	0.87 (0.53-1.42)	NA	NA	1.17 (0.62-2.21)
HOVON-126[15]	Ixa (6)	Pbo/Obs (6)	Ixa (4)	Pbo/Obs (6)	NA	NA	NA	NA
TOURMALINE-MM4[16]	Ixa (150)	Pbo/Obs (91)	Ixa (101)	Pbo/Obs (72)	0.765 (0.550-1.063)	NA	NA	NA
CASSIOPEIA[17]	Dara (57)	Pbo/Obs (70)	Dara (19)	Pbo/Obs (70)	0.43 (0.25-0.73)	NA	NA	NA
FORTE[18]	CarLen (39)	Len (44)	CarLen (14)	Len (22)	0.67 (0.34-1.31)	NA	NA	NA
TOURMALINE-MM2[19]	IxaLen (134)	Len (146)	IxaLen (80)	Len (104)	0.690 (0.506-0.941)	NA	NA	NA
RV-MM-PI-0752[20]	Len (13)	LenDex (17)	NA	NA	1.10 (0.47-2.55)	NA	NA	NA
MAIA[21]	DaraLenDex (48)	LenDex (44)	DaraLenDex (25)	LenDex (30)	0.55 (0.32-0.94)	DaraLenDex (25)	LenDex (26)	0.80 (0.46-1.39)
GRIFFIN[22]	DaraLen (42)	Len (37)	DaraLen (7)	Len (10)	0.38 (0.14-1.01)	NA	NA	NA
ATLAS[23]	CarLenDex (21)	Len (18)	CarLenDex (10)	Len (9)	0·74 (0·30–1·86)	NA	NA	NA
GEM2014MAIN[24]	LenDex (33)	IxaLenDex (31)	NA	NA	1.259 (0.641-2.500)	NA	NA	NA
PERSEUS[25]	DaraLen (76)	Len (78)	DaraLen (24)	Len (38)	0.59 (0.36–0.99)	NA	NA	NA

Abbreviations: *N.pts* Number of patients, *N.events* Number of events, *PFS* Progression-free survival, *OS* Overall survival, *HR* Hazard ratio, *CI* confidence interval, C*arLenDex* Carfilzomib, lenalidomide plus dexamethasone, *CarLen* Carfilzomib plus lenalidomide, *DaraLenDex* Daratumumab, lenalidomide plus dexamethasone, *DaraLen* Daratumumab plus lenalidomide, *IxaLenDex* Ixazomib, lenalidomide plus dexamethasone, *IxaLen* Ixazomib plus dexamethasone, *LenPdn* Lenalidomide plus prednisone, *LenDex* Lenalidomide plus dexamethasone, *Ixa* Ixazomib, *Len* Lenalidomide, *Dara* Daratumumab, *Pbo/Obs* Placebo or observation, *NA* not available.

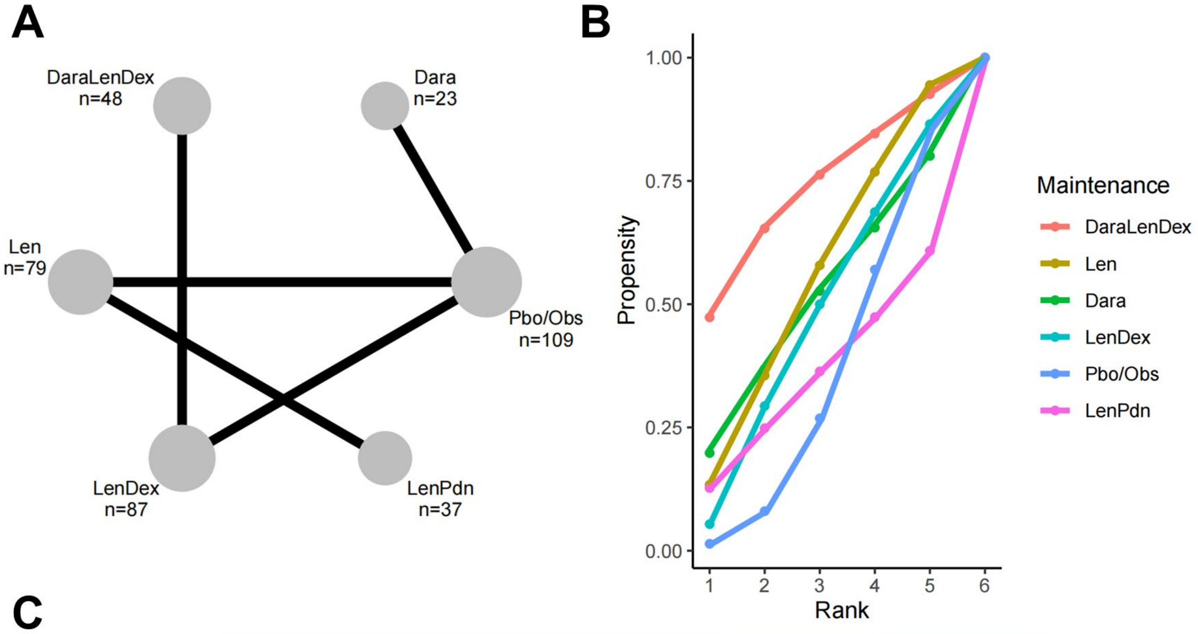

C

**DaraLenDex**	1.207 (0.52, 2.798)	1.233 (0.484, 3.134)	1.249 (0.721, 2.166)	1.356 (0.66, 2.779)	1.409 (0.493, 4.055)
0.829 (0.357, 1.924)	**Len**	1.022 (0.489, 2.135)	1.035 (0.545, 1.961)	1.123 (0.724, 1.736)	1.169 (0.623, 2.199)
0.811 (0.319, 2.066)	0.979 (0.468, 2.044)	**Dara**	1.012 (0.479, 2.151)	1.099 (0.609, 1.995)	1.144 (0.434, 3.017)
0.8 (0.462, 1.387)	0.966 (0.51, 1.834)	0.988 (0.465, 2.087)	**LenDex**	1.085 (0.683, 1.717)	1.128 (0.458, 2.764)
0.737 (0.36, 1.515)	0.891 (0.576, 1.38)	0.91 (0.501, 1.642)	0.922 (0.582, 1.464)	**Pbo/Obs**	1.04 (0.481, 2.241)
0.71 (0.247, 2.028)	0.855 (0.455, 1.605)	0.874 (0.331, 2.306)	0.886 (0.362, 2.181)	0.961 (0.446, 2.079)	**LenPdn**

Supplemental Figure 2. Results of OS by the analysis of HR

A) Network plot showing comparisons of overall survival (OS) by hazard ratio (HR); **B)** Cumulative ranking plot showing the surface under the cumulative ranking curve (SUCRA) for cytogenetic high-risk newly-diagnosed multiple myeloma (NDMM) patients; **C)** League table of commutatively comparative efficacy of different maintenance therapies. *Dex* Dexamethasone, *Len* Lenalidomide, *Dara* Daratumumab, *Pdn* Prednisone, *Pbo/Obs* Placebo or observation.

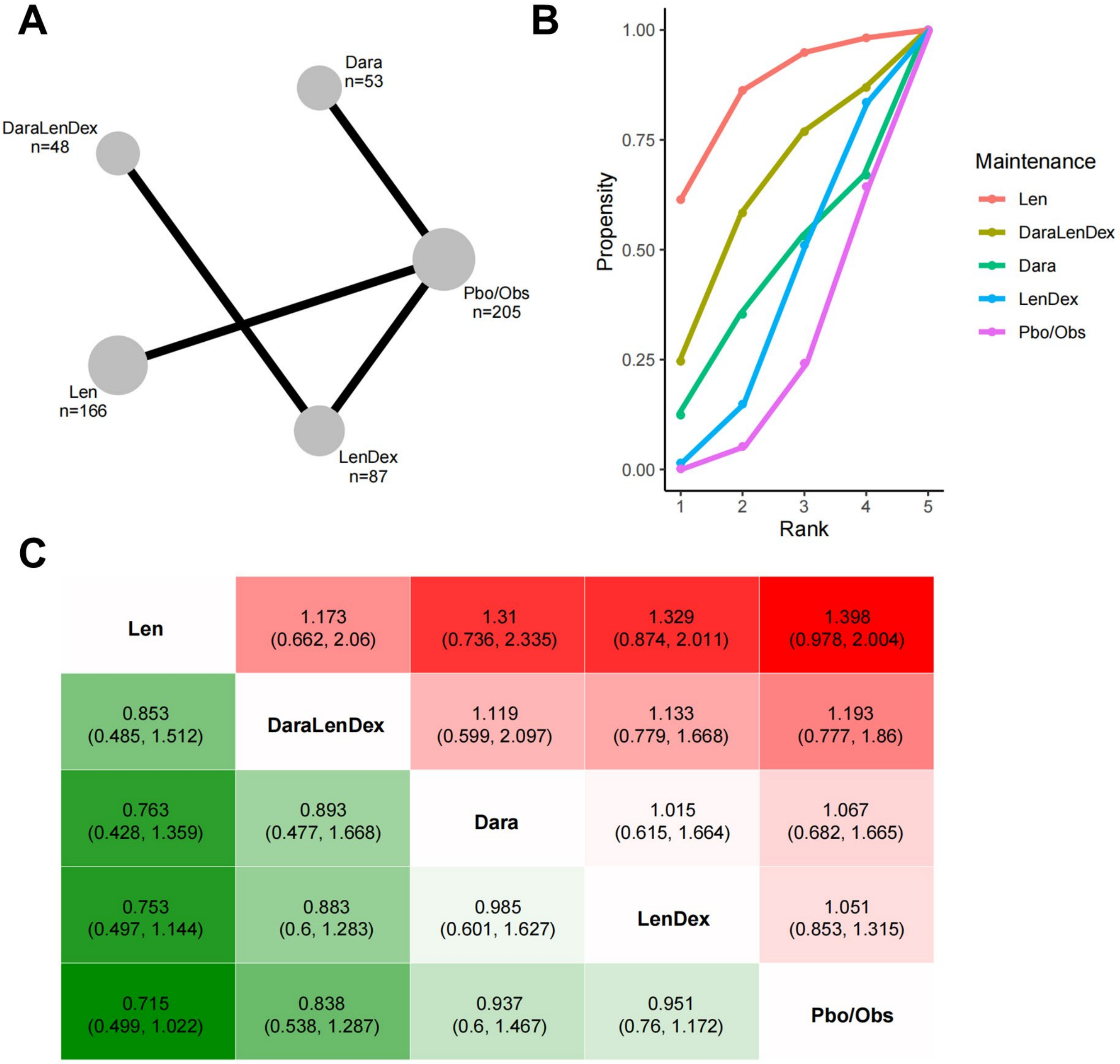

C

**Len**	1.173 (0.662, 2.06)	1.31 (0.736, 2.335)	1.329 (0.874, 2.011)	1.398 (0.978, 2.004)
0.853 (0.485, 1.512)	**DaraLenDex**	1.119 (0.599, 2.097)	1.133 (0.779, 1.668)	1.193 (0.777, 1.86)
0.763 (0.428, 1.359)	0.893 (0.477, 1.668)	**Dara**	1.015 (0.615, 1.664)	1.067 (0.682, 1.665)
0.753 (0.497, 1.144)	0.883 (0.6, 1.283)	0.985 (0.601, 1.627)	**LenDex**	1.051 (0.853, 1.315)
0.715 (0.499, 1.022)	0.838 (0.538, 1.287)	0.937 (0.6, 1.467)	0.951 (0.76, 1.172)	**Pbo/Obs**

Supplemental Figure 3. Results of OS by the analysis of RR

A) Network plot showing comparisons of overall survival (OS) by risk ratio (RR); **B)** Cumulative ranking plot showing the surface under the cumulative ranking curve (SUCRA) for cytogenetic high-risk newly-diagnosed multiple myeloma (NDMM) patients; **C)** League table of commutatively comparative efficacy of different maintenance therapies. *Dex* Dexamethasone, *Len* Lenalidomide, *Dara* Daratumumab, *Pbo/Obs* Placebo or observation.

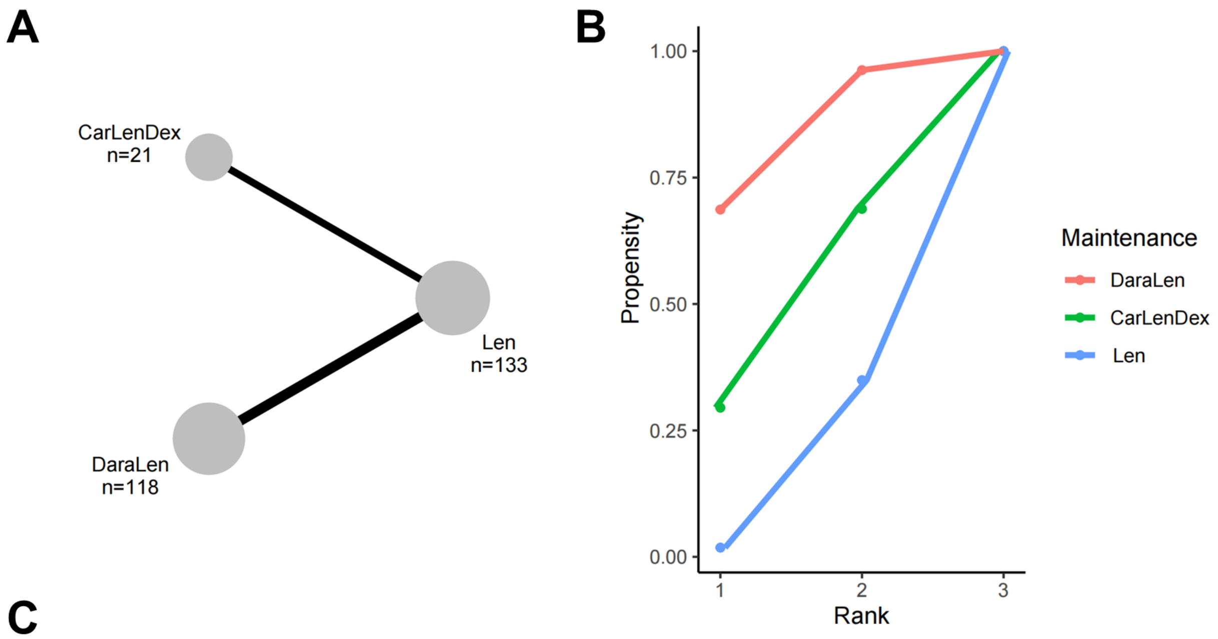

C

DaraLen	1.454 (0.293, 7.821)	1.948 (0.827, 5.077)
0.688 (0.128, 3.411)	CarLenDex	1.345 (0.343, 5.248)
0.513 (0.197, 1.208)	0.744 (0.191, 2.916)	Len

Supplemental Figure 4. Results of PFS for patients after ASCT by the analysis of HR

A) Network plot showing comparisons of progression-free survival (PFS) by hazard ratio (HR); **B)** Cumulative ranking plot showing the surface under the cumulative ranking curve (SUCRA) for cytogenetic high-risk newly-diagnosed multiple myeloma (NDMM) patients after autologous stem cell transplant (ASCT); **C)** League table of commutatively comparative efficacy of different maintenance therapies. *Dex* Dexamethasone, *Dara* Daratumumab, *Car* Carfilzomib, *Len* lenalidomide, *Pbo/Obs* Placebo or observation.

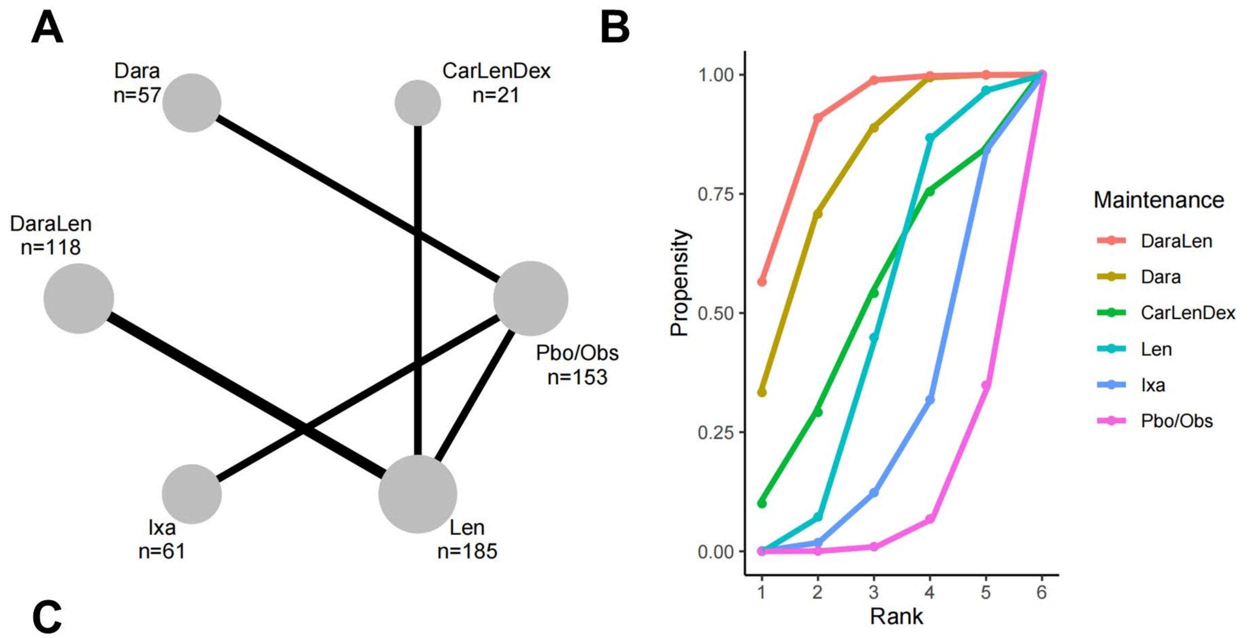

C

**DaraLen**	1.137 (0.544, 2.315)	1.504 (0.698, 3.303)	1.556 (1.098, 2.275)	1.978 (1.033, 3.786)	2.231 (1.237, 4.035)
0.879 (0.432, 1.838)	**Dara**	1.32 (0.541, 3.419)	1.373 (0.749, 2.602)	1.737 (1.076, 2.907)	1.956 (1.324, 3.06)
0.665 (0.303, 1.432)	0.757 (0.293, 1.85)	**CarLenDex**	1.041 (0.517, 2.042)	1.318 (0.546, 3.103)	1.487 (0.642, 3.351)
0.643 (0.44, 0.911)	0.728 (0.384, 1.335)	0.961 (0.49, 1.933)	**Len**	1.269 (0.737, 2.154)	1.428 (0.894, 2.265)
0.505 (0.264, 0.968)	0.576 (0.344, 0.929)	0.759 (0.322, 1.832)	0.788 (0.464, 1.356)	**Ixa**	1.127 (0.861, 1.484)
0.448 (0.248, 0.809)	0.511 (0.327, 0.755)	0.672 (0.298, 1.557)	0.7 (0.442, 1.119)	0.887 (0.674, 1.162)	**Pbo/Obs**

Supplemental Figure 5. Results of PFS for patients after ASCT by the analysis of RR

Network plot showing comparisons of progression-free survival (PFS) by risk ratio (RR); **B)** Cumulative ranking plot showing the surface under the cumulative ranking curve (SUCRA) for cytogenetic high-risk newly-diagnosed multiple myeloma (NDMM) patients after autologous stem cell transplant (ASCT); **C)** League table of commutatively comparative efficacy of different maintenance

therapies. *Dex* Dexamethasone, *Dara* Daratumumab, *Ixa* Ixazomib, *Car* Carfilzomib, *Len* lenalidomide, *Pbo/Obs* Placebo or observation.

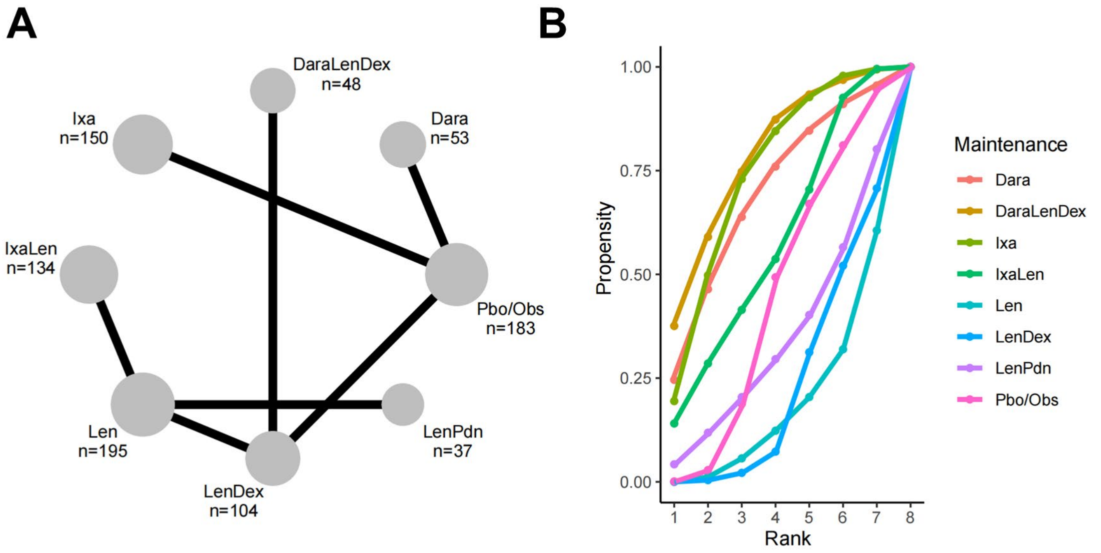

C

**DaraLenDex**	1.094 (0.507, 2.385)	1.117 (0.445, 2.827)	1.378 (0.482, 3.97)	1.431 (0.709, 2.893)	1.74 (0.567, 5.368)	**1.817 (1.057, 3.118)**	1.999 (0.73, 5.494)
0.914 (0.419, 1.972)	**Ixa**	1.017 (0.513, 2.017)	1.258 (0.438, 3.631)	1.306 (0.94, 1.814)	1.591 (0.514, 4.923)	1.656 (0.947, 2.889)	1.828 (0.664, 5.029)
0.896 (0.354, 2.249)	0.983 (0.496, 1.95)	**Dara**	1.234 (0.381, 3.969)	1.283 (0.702, 2.339)	1.56 (0.454, 5.379)	1.629 (0.763, 3.457)	1.791 (0.577, 5.545)
0.725 (0.252, 2.075)	0.795 (0.275, 2.285)	0.81 (0.252, 2.623)	**IxaLen**	1.038 (0.378, 2.848)	1.266 (0.703, 2.264)	1.32 (0.534, 3.224)	**1.451 (1.062, 1.978)**
0.699 (0.346, 1.411)	0.766 (0.551, 1.064)	0.779 (0.427, 1.425)	0.963 (0.351, 2.648)	**Pbo/Obs**	1.216 (0.414, 3.576)	1.269 (0.806, 1.992)	1.399 (0.535, 3.667)
0.575 (0.186, 1.764)	0.629 (0.203, 1.945)	0.641 (0.186, 2.204)	0.79 (0.442, 1.422)	0.823 (0.28, 2.416)	**LenPdn**	1.041 (0.393, 2.793)	1.148 (0.702, 1.883)
**0.55 (0.321, 0.946)**	0.604 (0.346, 1.056)	0.614 (0.289, 1.31)	0.758 (0.31, 1.873)	0.788 (0.502, 1.241)	0.96 (0.358, 2.542)	**LenDex**	1.102 (0.475, 2.574)
0.5 (0.182, 1.369)	0.547 (0.199, 1.507)	0.558 (0.18, 1.732)	**0.689 (0.505, 0.942)**	0.715 (0.273, 1.868)	0.871 (0.531, 1.425)	0.908 (0.388, 2.106)	**Len**

Supplemental Figure 6. Results of PFS for patients without ASCT by the analysis of HR

A) Network plot showing comparisons of progression-free survival (PFS) by hazard ratio (HR); **B)** Cumulative ranking plot showing the surface under the cumulative ranking curve (SUCRA) for cytogenetic high-risk newly-diagnosed multiple myeloma (NDMM) patients without autologous stem cell transplant (ASCT); **C)** League table of commutatively comparative efficacy of different maintenance therapies. *Dex* Dexamethasone, *Dara* Daratumumab, *Ixa* Ixazomib, *Car* Carfilzomib, *Len* lenalidomide, *Pdn* Prednisone, *Pbo/Obs* Placebo or observation.

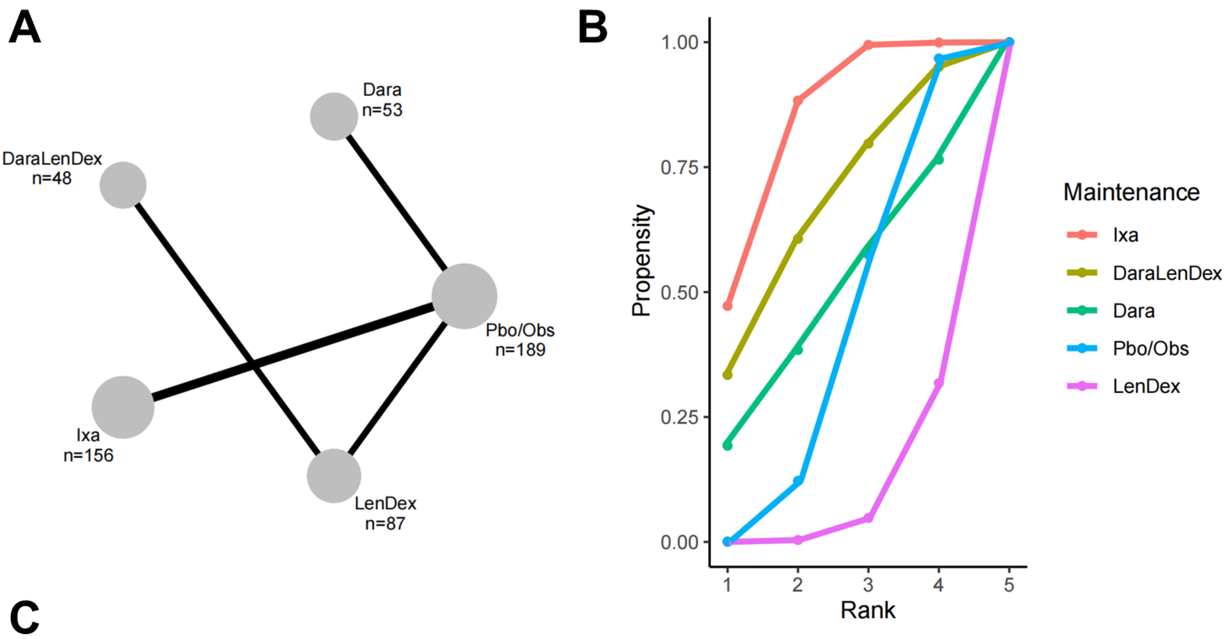

C

**Ixa**	1.064 (0.694, 1.615)	1.172 (0.734, 1.875)	**1.204 (1.034, 1.401)**	**1.386 (1.095, 1.779)**
0.94 (0.619, 1.442)	**DaraLenDex**	1.104 (0.611, 2.008)	1.133 (0.766, 1.692)	1.304 (0.93, 1.867)
0.853 (0.533, 1.362)	0.906 (0.498, 1.637)	**Dara**	1.027 (0.658, 1.599)	1.183 (0.731, 1.921)
**0.831 (0.714, 0.967)**	0.883 (0.591, 1.305)	0.974 (0.626, 1.52)	**Pbo/Obs**	1.15 (0.962, 1.408)
**0.722 (0.562, 0.914)**	0.767 (0.536, 1.075)	0.845 (0.52, 1.369)	0.87 (0.71, 1.04)	**LenDex**

Supplemental Figure 7. Results of PFS for patients without ASCT by the analysis of RR

A) Network plot showing comparisons of progression-free survival (PFS) by risk ratio (RR); **B)** Cumulative ranking plot showing the surface under the cumulative ranking curve (SUCRA) for cytogenetic high-risk newly-diagnosed multiple myeloma (NDMM) patients without autologous stem cell transplant (ASCT); **C)** League table of commutatively comparative efficacy of different maintenance therapies. *Dex* Dexamethasone, *Dara* Daratumumab, *Ixa* Ixazomib, *Len* lenalidomide, *Pbo/Obs* Placebo or observation.

Appendix 4: Heterogeneity Test and Funnel Plot

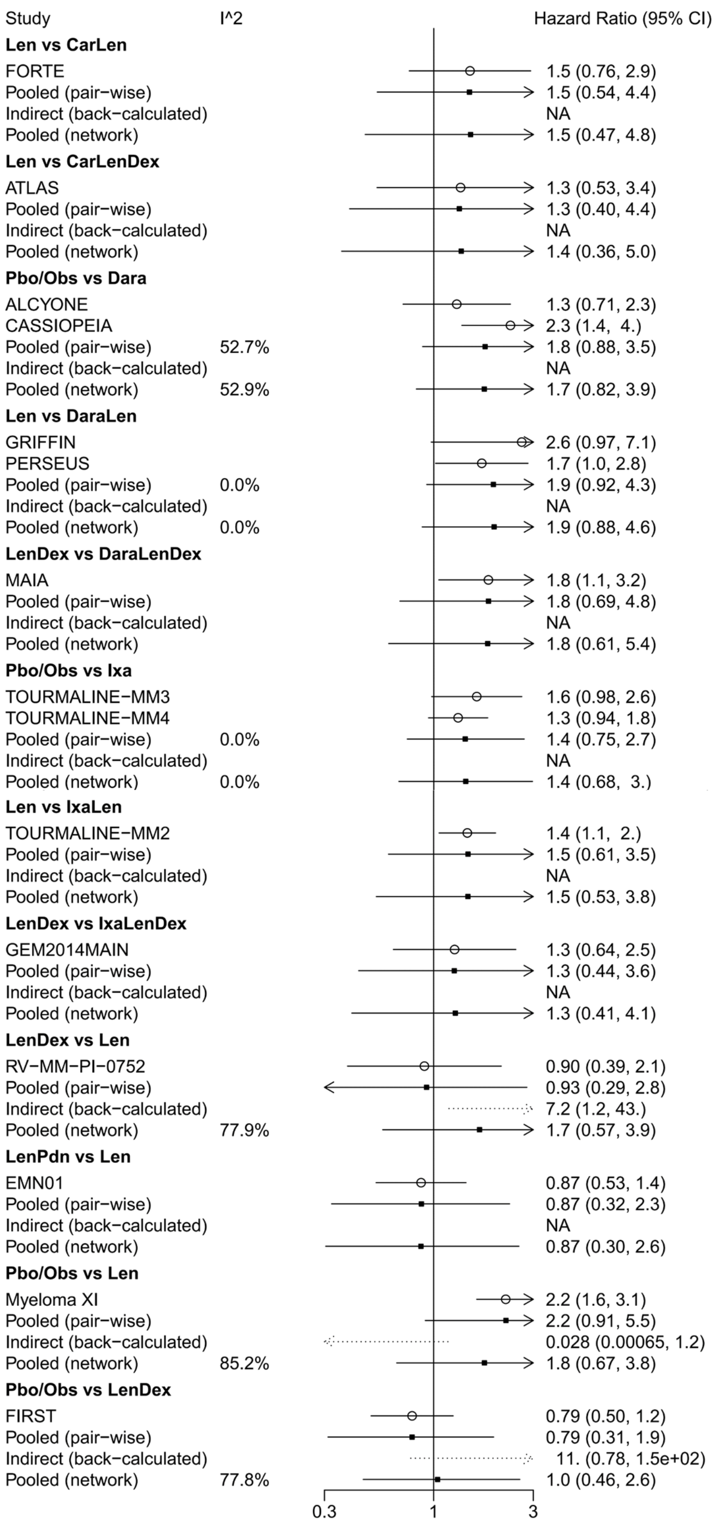

Supplemental Figure 8. Heterogeneity test of different maintenance therapies based on HR of PFS for high-risk NDMM patients

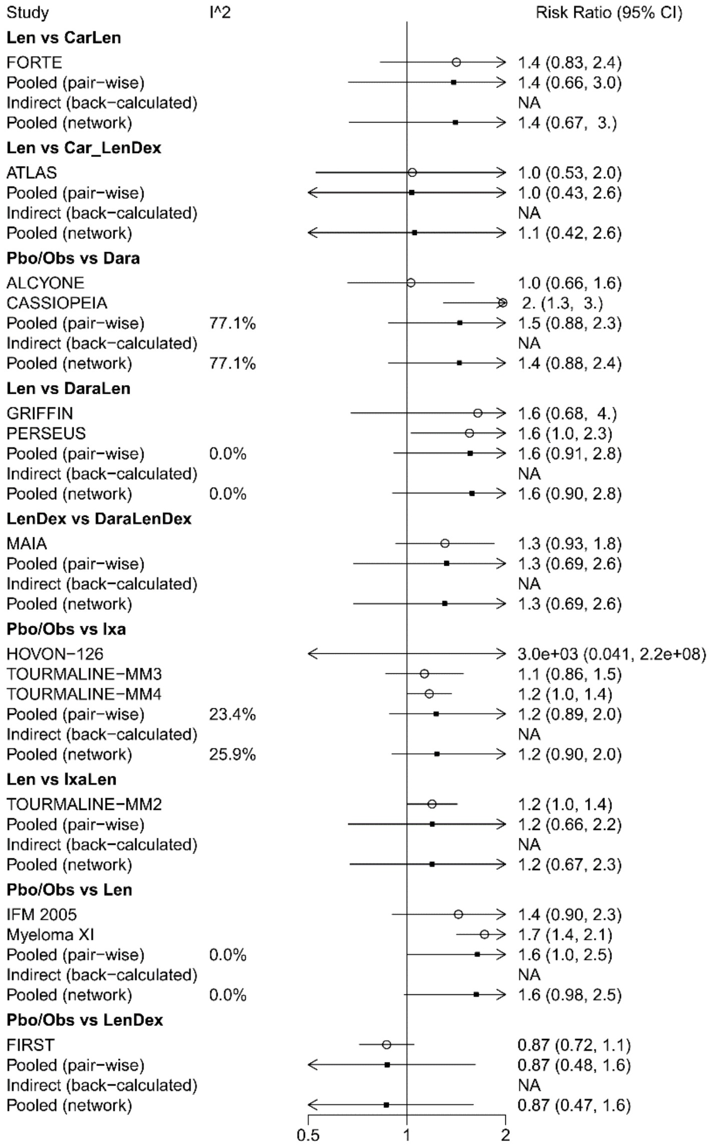

Supplemental Figure 9. Heterogeneity test of different maintenance therapies based on RR of PFS for high-risk NDMM patients without ASCT

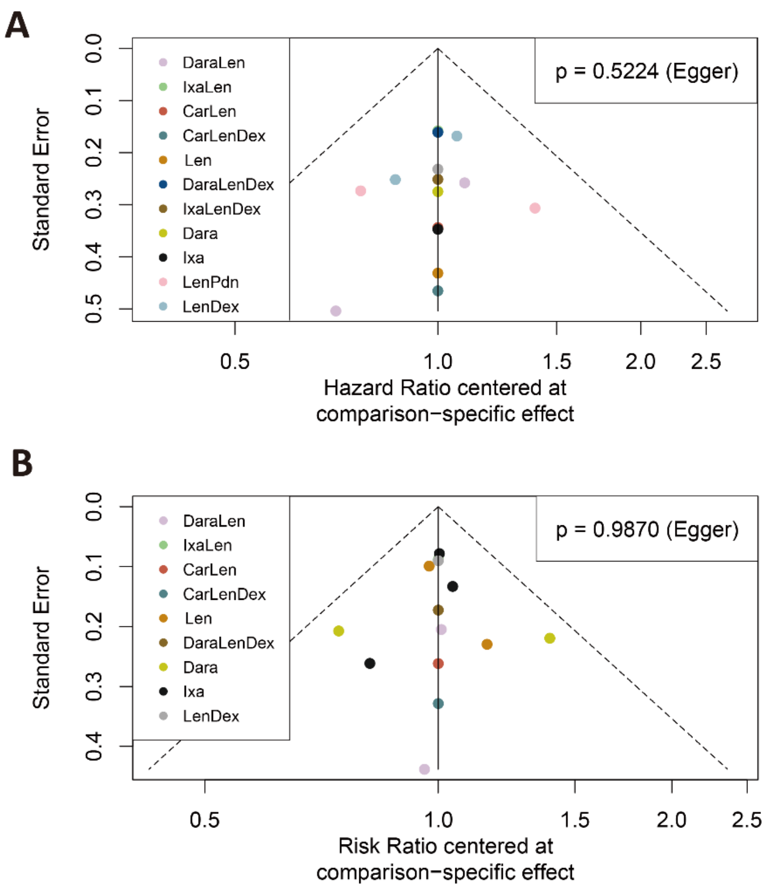

Supplemental Figure 10. Funnel plot of different maintenance therapies of PFS for high-risk NDMM patients.

(A) Publication bias of hazard ratio (HR) and **(B)** risk ratio (RR). *CarLenDex* Carfilzomib, lenalidomide plus dexamethasone; *CarLen* Carfilzomib plus lenalidomide; *Dara* Daratumumab; *DaraLen* Daratumumab plus lenalidomide; *DaraLenDex* Daratumumab, lenalidomide plus dexamethasone; *Ixa* Ixazomib; *IxaLen* Ixazomib plus lenalidomide; *IxaLenDex* Ixazomib, lenalidomide plus dexamethasone; *Len* Lenalidomide; *LenPdn* Lenalidomide plus prednisone; *LenDex* Lenalidomide plus dexamethasone.

Appendix 5: Rationale for the RoB 2 Assessment

Year	2012 Study ID IFM 2005		
**Domain**	**Signalling question**	**Response**	**Comments**
**Bias arising from the randomization process**	1.1 Was the allocation sequence random?	PY	Absence of specific information about generation of the randomization sequence, run by an experienced clinical trials unit; control group received placebo.
	1.2 Was the allocation sequence concealed until participants were enrolled and assigned to interventions?	Y	
	1.3 Did baseline differences between intervention groups suggest a problem with the randomization process?	PN	Balanced group size; adverse cytogenetic profiles, including the t(4;14) and the 17p deletion, were more common in the lenalidomide group (P=0.006).
	**Risk of bias judgement**	**Low**	
**Bias due to deviations from intended interventions**	2.1.Were participants aware of their assigned intervention during the trial?	N	Double blinding (participant, investigator).
	2.2.Were carers and people delivering the interventions aware of participants' assigned intervention during the trial?	N	
	2.3. If Y/PY/NI to 2.1 or 2.2: Were there deviations from the intended intervention that arose because of the experimental context?	*NA*	
	2.4 If Y/PY to 2.3: Were these deviations likely to have affected the outcome?	*NA*	
	2.5. If Y/PY/NI to 2.4: Were these deviations from intended intervention balanced between groups?	*NA*	
	2.6 Was an appropriate analysis used to estimate the effect of assignment to intervention?	Y	Analysis base on ITT.
	2.7 If N/PN/NI to 2.6: Was there potential for a substantial impact (on the result) of the failure to analyse participants in the group to which they were randomized?	*NA*	
	**Risk of bias judgement**	**Low**	
**Bias due to missing outcome data**	3.1 Were data for this outcome available for all, or nearly all, participants randomized?	PN	Six patients (1 in the Len group and 5 in the Pbo group) did not receive the assigned study drug. Thirty-seven patients (16 in the Len group and 21 in the Pbo group) did not receive consolidation treatment before maintenance therapy.
	3.2 If N/PN/NI to 3.1: Is there evidence that result was not biased by missing outcome data?	N	No bias correction or sensitivity analysis.
	3.3 If N/PN to 3.2: Could missingness in the outcome depend on its true value?	PY	Poor health status may lead to loss of follow up; balanced missingness between two groups.
	3.4 If Y/PY/NI to 3.3: Is it likely that missingness in the outcome depended on its true value?	N	
	**Risk of bias judgement**	**Some concerns**	
**Bias in measurement of the outcome**	4.1 Was the method of measuring the outcome inappropriate?	N	Treatment responses and disease progression were assessed according to the International Uniform Response Criteria for Multiple Myeloma.
	4.2 Could measurement or ascertainment of the outcome have differed between intervention groups?	N	Same measurement methods between groups.
	4.3 Were outcome assessors aware of the intervention received by study participants?	PN	The sponsor (Toulouse Hospital) collected the data and performed the final analysis in collaboration with the senior academic authors and an independent data and safety monitoring committee.
	4.4 If Y/PY/NI to 4.3: Could assessment of the outcome have been influenced by knowledge of intervention received?	*NA*	
	4.5 If Y/PY/NI to 4.4: Is it likely that assessment of the outcome was influenced by knowledge of intervention received?	*NA*	
	**Risk of bias judgement**	**Low**	
**Bias in selection of the reported result**	5.1 Were the data that produced this result analysed in accordance with a pre-specified analysis plan that was finalized before unblinded outcome data were available for analysis?	PY	Pre-specified intentions are available in ClinicalTrials.gov; last update were posted in April, 2019.
	5.2 ... multiple eligible outcome measurements (e.g. scales, definitions, time points) within the outcome domain?	N	The evaluation criteria of MM are consistent.
	5.3 ... multiple eligible analyses of the data?	PN	Reported results for the outcome measurement correspond to intended analyses according to the protocol.
	**Risk of bias judgement**	**Low**	

**Overall bias**	**Risk of bias judgement**	**Some concerns**	
**Year**	2018 **Study ID** TOURMALINE-MM3		
**Domain**	**Signalling question**	**Response**	**Comments**
**Bias arising from the randomization process**	1.1 Was the allocation sequence random?	PN	The randomisation scheme was generated by an independent statistician at the sponsor; patients, investigators, and study staff were blinded to treatment allocation.
	1.2 Was the allocation sequence concealed until participants were enrolled and assigned to interventions?	Y	
	1.3 Did baseline differences between intervention groups suggest a problem with the randomization process?	PY	A slightly higher proportion of younger patients and lower proportion of high-risk patients in the ixazomib versus placebo.
	**Risk of bias judgement**	**Some concerns**	
**Bias due to deviations from intended interventions**	2.1.Were participants aware of their assigned intervention during the trial?	N	Quadruple blinding (participant, care provider, investigator, outcomes assessor).
	2.2.Were carers and people delivering the interventions aware of participants' assigned intervention during the trial?	N	
	2.3. If Y/PY/NI to 2.1 or 2.2: Were there deviations from the intended intervention that arose because of the experimental context?	*NA*	
	2.4 If Y/PY to 2.3: Were these deviations likely to have affected the outcome?	*NA*	
	2.5. If Y/PY/NI to 2.4: Were these deviations from intended intervention balanced between groups?	*NA*	
	2.6 Was an appropriate analysis used to estimate the effect of assignment to intervention?	Y	Analysis based on ITT.
	2.7 If N/PN/NI to 2.6: Was there potential for a substantial impact (on the result) of the failure to analyse participants in the group to which they were randomized?	*NA*	
	**Risk of bias judgement**	**Low**	
**Bias due to missing outcome data**	3.1 Were data for this outcome available for all, or nearly all, participants randomized?	Y	Only 1 pt in Ixa group and 2 pts in Pbo group were not available for outcome assessment.
	3.2 If N/PN/NI to 3.1: Is there evidence that result was not biased by missing outcome data?	*NA*	
	3.3 If N/PN to 3.2: Could missingness in the outcome depend on its true value?	*NA*	
	3.4 If Y/PY/NI to 3.3: Is it likely that missingness in the outcome depended on its true value?	*NA*	
	**Risk of bias judgement**	**Low**	
**Bias in measurement of the outcome**	4.1 Was the method of measuring the outcome inappropriate?	N	Response and progressive disease assessments were based on central laboratory M-protein results, plus local bone marrow and imaging data, using IMWG 2011 criteria.
	4.2 Could measurement or ascertainment of the outcome have differed between intervention groups?	N	Same measurement between two groups.
	4.3 Were outcome assessors aware of the intervention received by study participants?	N	Outcomes were evaluated by an independent review committee blinded to both treatment assignment and investigator assessment of response.
	4.4 If Y/PY/NI to 4.3: Could assessment of the outcome have been influenced by knowledge of intervention received?	*NA*	
	4.5 If Y/PY/NI to 4.4: Is it likely that assessment of the outcome was influenced by knowledge of intervention received?	*NA*	
	**Risk of bias judgement**	**Low**	
**Bias in selection of the reported result**	5.1 Were the data that produced this result analysed in accordance with a pre-specified analysis plan that was finalized before unblinded outcome data were available for analysis?	Y	Consistent results analysis with detailed protocol.
	5.2 ... multiple eligible outcome measurements (e.g. scales, definitions, time points) within the outcome domain?	N	Measurements with recognised criteria, IMWG 2011.
	5.3 ... multiple eligible analyses of the data?	N	No evidence of multiple analysis methods.
	**Risk of bias judgement**	**Low**	
**Overall bias**	**Risk of bias judgement**	**Some concerns**	
**Year**	2018 **Study ID** MYELOMA XI		
**Domain**	**Signalling question**	**Response**	**Comments**
	1.1 Was the allocation sequence random?	Y	

**Bias arising from the randomization process**	1.2 Was the allocation sequence concealed until participants were enrolled and assigned to interventions?	Y	A computer-generated minimisation algorithm was used; all randomisations were done at the Clinical Trials Research Unit (Leeds, UK) by authorised members of staff with a centralised automated 24h telephone system.
	1.3 Did baseline differences between intervention groups suggest a problem with the randomization process?	N	Patient and disease characteristics were well balanced between groups.
	**Risk of bias judgement**	**Low**	
**Bias due to deviations from intended interventions**	2.1.Were participants aware of their assigned intervention during the trial?	Y	Open-label trial.
	2.2.Were carers and people delivering the interventions aware of participants' assigned intervention during the trial?	Y	
	2.3. If Y/PY/NI to 2.1 or 2.2: Were there deviations from the intended intervention that arose because of the experimental context?	NI	Not reported.
	2.4 If Y/PY to 2.3: Were these deviations likely to have affected the outcome?	*NA*	
	2.5. If Y/PY/NI to 2.4: Were these deviations from intended intervention balanced between groups?	*NA*	
	2.6 Was an appropriate analysis used to estimate the effect of assignment to intervention?	Y	Analysis based on ITT.
	2.7 If N/PN/NI to 2.6: Was there potential for a substantial impact (on the result) of the failure to analyse participants in the group to which they were randomized?	*NA*	
	**Risk of bias judgement**	**Some concerns**	
**Bias due to missing outcome data**	3.1 Were data for this outcome available for all, or nearly all, participants randomized?	Y	Only 5 missingness in Len group.
	3.2 If N/PN/NI to 3.1: Is there evidence that result was not biased by missing outcome data?	*NA*	
	3.3 If N/PN to 3.2: Could missingness in the outcome depend on its true value?	*NA*	
	3.4 If Y/PY/NI to 3.3: Is it likely that missingness in the outcome depended on its true value?	*NA*	
	**Risk of bias judgement**	**Low**	
**Bias in measurement of the outcome**	4.1 Was the method of measuring the outcome inappropriate?	N	Recognised measurement methods.
	4.2 Could measurement or ascertainment of the outcome have differed between intervention groups?	N	Same measurement between groups.
	4.3 Were outcome assessors aware of the intervention received by study participants?	N	Response and disease progression were assessed on the basis of IMWG Uniform Response criteria and reviewed centrally by an expert panel masked to treatment allocation.
	4.4 If Y/PY/NI to 4.3: Could assessment of the outcome have been influenced by knowledge of intervention received?	*NA*	
	4.5 If Y/PY/NI to 4.4: Is it likely that assessment of the outcome was influenced by knowledge of intervention received?	*NA*	
	**Risk of bias judgement**	**Low**	
**Bias in selection of the reported result**	5.1 Were the data that produced this result analysed in accordance with a pre-specified analysis plan that was finalized before unblinded outcome data were available for analysis?	Y	Comformed with pre-specified analysis plan.
	5.2 ... multiple eligible outcome measurements (e.g. scales, definitions, time points) within the outcome domain?	N	Outcomes were measured in an objective manner.
	5.3 ... multiple eligible analyses of the data?	N	Recognised analytical methods were used.
	**Risk of bias judgement**	**Low**	
**Overall bias**	**Risk of bias judgement**	**Some concerns**	
**Year**	2018 **Study ID** FIRST		
**Domain**	**Signalling question**	**Response**	**Comments**
**Bias arising from the randomization process**	1.1 Was the allocation sequence random?	NI	The only information about randomization methods is a statement that the study is randomized.
	1.2 Was the allocation sequence concealed until participants were enrolled and assigned to interventions?	NI	
	1.3 Did baseline differences between intervention groups suggest a problem with the randomization process?	N	Baseline characteristics were well balanced.
	**Risk of bias judgement**	**Some concerns**	

Bias due to deviations from intended interventions	2.1.Were participants aware of their assigned intervention during the trial?	Y	Open-label trial.
	2.2.Were carers and people delivering the interventions aware of participants' assigned intervention during the trial?	Y	
	2.3. If Y/PY/NI to 2.1 or 2.2: Were there deviations from the intended intervention that arose because of the experimental context?	NI	Not reported.
	2.4 If Y/PY to 2.3: Were these deviations likely to have affected the outcome?	*NA*	
	2.5. If Y/PY/NI to 2.4: Were these deviations from intended intervention balanced between groups?	*NA*	
	2.6 Was an appropriate analysis used to estimate the effect of assignment to intervention?	Y	Based on ITT.
	2.7 If N/PN/NI to 2.6: Was there potential for a substantial impact (on the result) of the failure to analyse participants in the group to which they were randomized?	*NA*	
	**Risk of bias judgement**	**Some concerns**	
**Bias due to missing outcome data**	3.1 Were data for this outcome available for all, or nearly all, participants randomized?	PY	Over 95% pts were evaluated.
	3.2 If N/PN/NI to 3.1: Is there evidence that result was not biased by missing outcome data?	*NA*	
	3.3 If N/PN to 3.2: Could missingness in the outcome depend on its true value?	*NA*	
	3.4 If Y/PY/NI to 3.3: Is it likely that missingness in the outcome depended on its true value?	*NA*	
	**Risk of bias judgement**	**Low**	
**Bias in measurement of the outcome**	4.1 Was the method of measuring the outcome inappropriate?	N	Response to treatment was evaluated using the International Myeloma Working Group criteria for multiple myeloma and was assessed after each treatment cycle and every 28 days during follow-up.
	4.2 Could measurement or ascertainment of the outcome have differed between intervention groups?	N	Same evaluation between groups.
	4.3 Were outcome assessors aware of the intervention received by study participants?	Y	Not evidence of blinding.
	4.4 If Y/PY/NI to 4.3: Could assessment of the outcome have been influenced by knowledge of intervention received?	PN	Assessment of the outcome needed to be supported by objective laboratory findings.
	4.5 If Y/PY/NI to 4.4: Is it likely that assessment of the outcome was influenced by knowledge of intervention received?	*NA*	
	**Risk of bias judgement**	**Low**	
**Bias in selection of the reported result**	5.1 Were the data that produced this result analysed in accordance with a pre-specified analysis plan that was finalized before unblinded outcome data were available for analysis?	Y	Conformed with protocol.
	5.2 ... multiple eligible outcome measurements (e.g. scales, definitions, time points) within the outcome domain?	N	Uniform criteria for judging progression and death.
	5.3 ... multiple eligible analyses of the data?	N	No multiple eligible analyses.
	**Risk of bias judgement**	**Low**	
**Overall bias**	**Risk of bias judgement**	**Some concerns**	

**Year**	2019 **Study ID** ALCYONE		
**Domain**	**Signalling question**	**Response**	**Comments**
**Bias arising from the randomization process**	1.1 Was the allocation sequence random?	Y	Permuted block randomisation; an interactive web-based randomisation system (IRT, Signant Health, Wayne, PA, USA) was used, and each patient was assigned a unique number.
	1.2 Was the allocation sequence concealed until participants were enrolled and assigned to interventions?	Y	
	1.3 Did baseline differences between intervention groups suggest a problem with the randomization process?	N	Patient demographics and baseline disease characteristics were well balanced between groups.
	**Risk of bias judgement**	**Low**	
**Bias due to deviations from intended interventions**	2.1.Were participants aware of their assigned intervention during the trial?	Y	No masking.
	2.2.Were carers and people delivering the interventions aware of participants' assigned intervention during the trial?	Y	
	2.3. If Y/PY/NI to 2.1 or 2.2: Were there deviations from the intended intervention that arose because of the experimental context?	NI	Not reported.
	2.4 If Y/PY to 2.3: Were these deviations likely to have affected the outcome?	*NA*	
	2.5. If Y/PY/NI to 2.4: Were these deviations from intended intervention balanced between groups?	*NA*	

	2.6 Was an appropriate analysis used to estimate the effect of assignment to intervention?	Y	Based on ITT.
	2.7 If N/PN/NI to 2.6: Was there potential for a substantial impact (on the result) of the failure to analyse participants in the group to which they were randomized?	*NA*	
	**Risk of bias judgement**	**Some concerns**	
**Bias due to missing outcome data**	3.1 Were data for this outcome available for all, or nearly all, participants randomized?	Y	No more than 5% pts were missing.
	3.2 If N/PN/NI to 3.1: Is there evidence that result was not biased by missing outcome data?	*NA*	
	3.3 If N/PN to 3.2: Could missingness in the outcome depend on its true value?	*NA*	
	3.4 If Y/PY/NI to 3.3: Is it likely that missingness in the outcome depended on its true value?	*NA*	
	**Risk of bias judgement**	**Low**	
**Bias in measurement of the outcome**	4.1 Was the method of measuring the outcome inappropriate?	N	International Myeloma Working Group criteria16 were used to define all efficacy responses, including progressive disease and negative status for minimal residual disease.
	4.2 Could measurement or ascertainment of the outcome have differed between intervention groups?	N	Same measurement.
	4.3 Were outcome assessors aware of the intervention received by study participants?	PY	Open-label trial.
	4.4 If Y/PY/NI to 4.3: Could assessment of the outcome have been influenced by knowledge of intervention received?	PN	Assessment of outcomes needed to meet the criteria for objective laboratory findings.
	4.5 If Y/PY/NI to 4.4: Is it likely that assessment of the outcome was influenced by knowledge of intervention received?	*NA*	
	**Risk of bias judgement**	**Low**	
**Bias in selection of the reported result**	5.1 Were the data that produced this result analysed in accordance with a pre-specified analysis plan that was finalized before unblinded outcome data were available for analysis?	Y	Conformed with protocol.
	5.2 ... multiple eligible outcome measurements (e.g. scales, definitions, time points) within the outcome domain?	N	Recognised assessment of outcomes.
	5.3 ... multiple eligible analyses of the data?	PN	Recognised analysis of outcomes; prespecified analyses by MRD status.
	**Risk of bias judgement**	**Low**	
**Overall bias**	**Risk of bias judgement**	**Some concerns**	

**Year**	2019 **Study ID** EMN01		
**Domain**	**Signalling question**	**Response**	**Comments**
**Bias arising from the randomization process**	1.1 Was the allocation sequence random?	Y	Randomized was based on a computer-generated randomization schedule prepared by the Coordinating Centre.
	1.2 Was the allocation sequence concealed until participants were enrolled and assigned to interventions?	PY	
	1.3 Did baseline differences between intervention groups suggest a problem with the randomization process?	N	Balanced baseline.
	**Risk of bias judgement**	**Low**	
**Bias due to deviations from intended interventions**	2.1.Were participants aware of their assigned intervention during the trial?	Y	No masking.
	2.2.Were carers and people delivering the interventions aware of participants' assigned intervention during the trial?	Y	
	2.3. If Y/PY/NI to 2.1 or 2.2: Were there deviations from the intended intervention that arose because of the experimental context?	NI	Not reported
	2.4 If Y/PY to 2.3: Were these deviations likely to have affected the outcome?	*NA*	
	2.5. If Y/PY/NI to 2.4: Were these deviations from intended intervention balanced between groups?	*NA*	
	2.6 Was an appropriate analysis used to estimate the effect of assignment to intervention?	Y	Based on ITT.
	2.7 If N/PN/NI to 2.6: Was there potential for a substantial impact (on the result) of the failure to analyse participants in the group to which they were randomized?	*NA*	
	**Risk of bias judgement**	**Some concerns**	
	3.1 Were data for this outcome available for all, or nearly all, participants randomized?	Y	Outcomes of all pts were available.
	3.2 If N/PN/NI to 3.1: Is there evidence that result was not biased by missing outcome data?	*NA*	

**Bias due to missing outcome data**	*3.3 If N/PN to 3.2: Could missingness in the outcome depend on its true value?*	*NA*	
	3.4 If Y/PY/NI to 3.3: Is it likely that missingness in the outcome depended on its true value?	*NA*	
	**Risk of bias judgement**	**Low**	
**Bias in measurement of the outcome**	4.1 Was the method of measuring the outcome inappropriate?	N	Evaluation of the response to the treatments was performed according to the International Response Criteria for Multiple Myeloma.
	4.2 Could measurement or ascertainment of the outcome have differed between intervention groups?	N	Same measurement.
	4.3 Were outcome assessors aware of the intervention received by study participants?	NI	No evidence of blinding.
	4.4 If Y/PY/NI to 4.3: Could assessment of the outcome have been influenced by knowledge of intervention received?	PN	Assessment of outcomes needed to meet the criteria for objective laboratory findings.
	4.5 If Y/PY/NI to 4.4: Is it likely that assessment of the outcome was influenced by knowledge of intervention received?	*NA*	
	**Risk of bias judgement**	**Low**	
**Bias in selection of the reported result**	5.1 Were the data that produced this result analysed in accordance with a pre-specified analysis plan that was finalized before unblinded outcome data were available for analysis?	Y	Consistent with protocol.
	5.2 ... multiple eligible outcome measurements (e.g. scales, definitions, time points) within the outcome domain?	N	Outcomes were assessed with recognized measurements.
	5.3 ... multiple eligible analyses of the data?	N	No multiple eligible analyses.
	**Risk of bias judgement**	**Low**	
**Overall bias**	**Risk of bias judgement**	**Some concerns**	

**Year**	2020 **Study ID** HOVON-126		
**Domain**	**Signalling question**	**Response**	**Comments**
**Bias arising from the randomization process**	1.1 Was the allocation sequence random?	NI	The only information about randomization methods is a statement that the study is randomized.
	1.2 Was the allocation sequence concealed until participants were enrolled and assigned to interventions?	NI	
	1.3 Did baseline differences between intervention groups suggest a problem with the randomization process?	PN	Almost balanced baseline, probably due to small sample size.
	**Risk of bias judgement**	**Some concerns**	
**Bias due to deviations from intended interventions**	2.1.Were participants aware of their assigned intervention during the trial?	Y	Open-label trial.
	2.2.Were carers and people delivering the interventions aware of participants' assigned intervention during the trial?	Y	
	2.3. If Y/PY/NI to 2.1 or 2.2: Were there deviations from the intended intervention that arose because of the experimental context?	PN	Changes to intervention that are consistent with the trial protocol.
	2.4 If Y/PY to 2.3: Were these deviations likely to have affected the outcome?	*NA*	
	2.5. If Y/PY/NI to 2.4: Were these deviations from intended intervention balanced between groups?	*NA*	
	2.6 Was an appropriate analysis used to estimate the effect of assignment to intervention?	Y	Based on ITT.
	2.7 If N/PN/NI to 2.6: Was there potential for a substantial impact (on the result) of the failure to analyse participants in the group to which they were randomized?	*NA*	
	**Risk of bias judgement**	**Low**	
**Bias due to missing outcome data**	3.1 Were data for this outcome available for all, or nearly all, participants randomized?	Y	All the pts were evaluated.
	3.2 If N/PN/NI to 3.1: Is there evidence that result was not biased by missing outcome data?	*NA*	
	3.3 If N/PN to 3.2: Could missingness in the outcome depend on its true value?	*NA*	
	3.4 If Y/PY/NI to 3.3: Is it likely that missingness in the outcome depended on its true value?	*NA*	
	**Risk of bias judgement**	**Low**	
**Bias in measurement of the outcome**	4.1 Was the method of measuring the outcome inappropriate?	PN	Based on IMWG criteria.
	4.2 Could measurement or ascertainment of the outcome have differed between intervention groups?	N	Same methods between groups.
	4.3 Were outcome assessors aware of the intervention received by study participants?	Y	No blinding.

	4.4 If Y/PY/NI to 4.3: Could assessment of the outcome have been influenced by knowledge of intervention received?	PN	The assessment of outcomes was based on objective laboratory tests.
	4.5 If Y/PY/NI to 4.4: Is it likely that assessment of the outcome was influenced by knowledge of intervention received?	*NA*	
	**Risk of bias judgement**	**Low**	
**Bias in selection of the reported result**	5.1 Were the data that produced this result analysed in accordance with a pre-specified analysis plan that was finalized before unblinded outcome data were available for analysis?	PY	Consistent with the pre-specified analysis plan.
	5.2 ... multiple eligible outcome measurements (e.g. scales, definitions, time points) within the outcome domain?	N	Few possible ways in which the outcome domain can be measured.
	5.3 ... multiple eligible analyses of the data?	N	Recognized outcome analysis.
	**Risk of bias judgement**	**Low**	
**Overall bias**	**Risk of bias judgement**	**Some concerns**	

**Year**	2020 **Study ID** TOURMALINE-MM4		
**Domain**	**Signalling question**	**Response**	**Comments**
**Bias arising from the randomization process**	1.1 Was the allocation sequence random?	Y	Centralized randomization through an interactive voice/web response system was employed; double blind trial.
	1.2 Was the allocation sequence concealed until participants were enrolled and assigned to interventions?	Y	
	1.3 Did baseline differences between intervention groups suggest a problem with the randomization process?	N	Baseline patient demographics and disease characteristics, including prior induction treatment, were well balanced between groups.
	**Risk of bias judgement**	**Low**	
**Bias due to deviations from intended interventions**	2.1.Were participants aware of their assigned intervention during the trial?	N	Quadruple masking (participant, care provider, investigator, outcomes assessor).
	2.2.Were carers and people delivering the interventions aware of participants' assigned intervention during the trial?	N	
	2.3. If Y/PY/NI to 2.1 or 2.2: Were there deviations from the intended intervention that arose because of the experimental context?	*NA*	
	2.4 If Y/PY to 2.3: Were these deviations likely to have affected the outcome?	*NA*	
	2.5. If Y/PY/NI to 2.4: Were these deviations from intended intervention balanced between groups?	*NA*	
	2.6 Was an appropriate analysis used to estimate the effect of assignment to intervention?	Y	Based on ITT.
	2.7 If N/PN/NI to 2.6: Was there potential for a substantial impact (on the result) of the failure to analyse participants in the group to which they were randomized?	*NA*	
	**Risk of bias judgement**	**Low**	
**Bias due to missing outcome data**	3.1 Were data for this outcome available for all, or nearly all, participants randomized?	Y	All the pts were available.
	3.2 If N/PN/NI to 3.1: Is there evidence that result was not biased by missing outcome data?	*NA*	
	3.3 If N/PN to 3.2: Could missingness in the outcome depend on its true value?	*NA*	
	3.4 If Y/PY/NI to 3.3: Is it likely that missingness in the outcome depended on its true value?	*NA*	
	**Risk of bias judgement**	**Low**	
**Bias in measurement of the outcome**	4.1 Was the method of measuring the outcome inappropriate?	N	Response and PD were evaluated by an IRC blinded to both treatment assignment and investigator assessment of response; assessments were based on central laboratory M-protein results, plus local bone marrow and imaging data, using IMWG 2011 criteria.
	4.2 Could measurement or ascertainment of the outcome have differed between intervention groups?	N	Same measurement between groups.
	4.3 Were outcome assessors aware of the intervention received by study participants?	N	Outcome assessors were blinded.
	4.4 If Y/PY/NI to 4.3: Could assessment of the outcome have been influenced by knowledge of intervention received?	*NA*	
	4.5 If Y/PY/NI to 4.4: Is it likely that assessment of the outcome was influenced by knowledge of intervention received?	*NA*	
	**Risk of bias judgement**	**Low**	
	5.1 Were the data that produced this result analysed in accordance with a pre-specified analysis plan that was finalized before unblinded outcome data were available for analysis?	Y	In accordance with protocol online.

**Bias in selection of the reported result**	5.2 ... multiple eligible outcome measurements (e.g. scales, definitions, time points) within the outcome domain?	N	All eligible reported results for the outcome domain correspond to all intended outcome measurements.
	5.3 ... multiple eligible analyses of the data?	N	All eligible reported results for the outcome measurement correspond to all intended analyses.
	**Risk of bias judgement**	**Low**	
**Overall bias**	**Risk of bias judgement**	**Low**	

**Year**	2021 **Study ID** CASSIOPEIA		
**Domain**	**Signalling question**	**Response**	**Comments**
**Bias arising from the randomization process**	1.1 Was the allocation sequence random?	Y	Research staff used an interactive web-based system, balanced using permuted blocks of four, to generate treatment assignments.
	1.2 Was the allocation sequence concealed until participants were enrolled and assigned to interventions?	Y	
	1.3 Did baseline differences between intervention groups suggest a problem with the randomization process?	N	Balanced baseline.
	**Risk of bias judgement**	**Low**	
**Bias due to deviations from intended interventions**	2.1.Were participants aware of their assigned intervention during the trial?	Y	No masking.
	2.2.Were carers and people delivering the interventions aware of participants' assigned intervention during the trial?	Y	
	2.3. If Y/PY/NI to 2.1 or 2.2: Were there deviations from the intended intervention that arose because of the experimental context?	N	Most of these protocol deviations were safety assessment deviations in the daratumumab group, including but not limited to events concerning daratumumab infusion.
	2.4 If Y/PY to 2.3: Were these deviations likely to have affected the outcome?	*NA*	
	2.5. If Y/PY/NI to 2.4: Were these deviations from intended intervention balanced between groups?	*NA*	
	2.6 Was an appropriate analysis used to estimate the effect of assignment to intervention?	Y	Based on ITT.
	2.7 If N/PN/NI to 2.6: Was there potential for a substantial impact (on the result) of the failure to analyse participants in the group to which they were randomized?	*NA*	
	**Risk of bias judgement**	**Some concerns**	
**Bias due to missing outcome data**	3.1 Were data for this outcome available for all, or nearly all, participants randomized?	Y	Only 2 pts in Dara group were missing.
	3.2 If N/PN/NI to 3.1: Is there evidence that result was not biased by missing outcome data?	*NA*	
	3.3 If N/PN to 3.2: Could missingness in the outcome depend on its true value?	*NA*	
	3.4 If Y/PY/NI to 3.3: Is it likely that missingness in the outcome depended on its true value?	*NA*	
	**Risk of bias judgement**	**Low**	
**Bias in measurement of the outcome**	4.1 Was the method of measuring the outcome inappropriate?	N	A central laboratory did the disease assessments once every 8 weeks after the second randomisation.
	4.2 Could measurement or ascertainment of the outcome have differed between intervention groups?	N	Same measurement.
	4.3 Were outcome assessors aware of the intervention received by study participants?	Y	
	4.4 If Y/PY/NI to 4.3: Could assessment of the outcome have been influenced by knowledge of intervention received?	N	Assessment of the outcome relied on objective laboratory findings.
	4.5 If Y/PY/NI to 4.4: Is it likely that assessment of the outcome was influenced by knowledge of intervention received?	*NA*	
	**Risk of bias judgement**	**Low**	
**Bias in selection of the reported result**	5.1 Were the data that produced this result analysed in accordance with a pre-specified analysis plan that was finalized before unblinded outcome data were available for analysis?	Y	In accordance with a pre-specified analysis plan.
	5.2 ... multiple eligible outcome measurements (e.g. scales, definitions, time points) within the outcome domain?	N	All eligible reported results for the outcome domain correspond to all intended outcome measurements.
	5.3 ... multiple eligible analyses of the data?	N	All eligible reported results for the outcome domain correspond to all intended outcome analysis.
	**Risk of bias judgement**	**Low**	
**Overall bias**	**Risk of bias judgement**	**Some concerns**	

Year	2021	Study ID	FORTE
**Domain**	**Signalling question**	**Response**	**Comments**
**Bias arising from the randomization process**	1.1 Was the allocation sequence random?	Y	A computer system was used for randomization.
	1.2 Was the allocation sequence concealed until participants were enrolled and assigned to interventions?	Y	
	1.3 Did baseline differences between intervention groups suggest a problem with the randomization process?	N	Balanced baseline.
	**Risk of bias judgement**	**Low**	
**Bias due to deviations from intended interventions**	2.1.Were participants aware of their assigned intervention during the trial?	Y	Open-label trial.
	2.2.Were carers and people delivering the interventions aware of participants' assigned intervention during the trial?	Y	
	2.3. If Y/PY/NI to 2.1 or 2.2: Were there deviations from the intended intervention that arose because of the experimental context?	NI	Not reported
	2.4 If Y/PY to 2.3: Were these deviations likely to have affected the outcome?	*NA*	
	2.5. If Y/PY/NI to 2.4: Were these deviations from intended intervention balanced between groups?	*NA*	
	2.6 Was an appropriate analysis used to estimate the effect of assignment to intervention?	Y	Based on ITT.
	2.7 If N/PN/NI to 2.6: Was there potential for a substantial impact (on the result) of the failure to analyse participants in the group to which they were randomized?	*NA*	
	**Risk of bias judgement**	**Some concerns**	
**Bias due to missing outcome data**	3.1 Were data for this outcome available for all, or nearly all, participants randomized?	Y	Over 95% pts were evaluable.
	3.2 If N/PN/NI to 3.1: Is there evidence that result was not biased by missing outcome data?	*NA*	
	3.3 If N/PN to 3.2: Could missingness in the outcome depend on its true value?	*NA*	
	3.4 If Y/PY/NI to 3.3: Is it likely that missingness in the outcome depended on its true value?	*NA*	
	**Risk of bias judgement**	**Low**	
**Bias in measurement of the outcome**	4.1 Was the method of measuring the outcome inappropriate?	N	The response rate was defined according to the International Uniform Response Criteria.
	4.2 Could measurement or ascertainment of the outcome have differed between intervention groups?	N	Same measurement.
	4.3 Were outcome assessors aware of the intervention received by study participants?	Y	No blinding.
	4.4 If Y/PY/NI to 4.3: Could assessment of the outcome have been influenced by knowledge of intervention received?	PY	Knowledge of intervention status could have influenced outcome assessment but there is no reason to believe that it did.
	4.5 If Y/PY/NI to 4.4: Is it likely that assessment of the outcome was influenced by knowledge of intervention received?	PN	
	**Risk of bias judgement**	**Some concerns**	
**Bias in selection of the reported result**	5.1 Were the data that produced this result analysed in accordance with a pre-specified analysis plan that was finalized before unblinded outcome data were available for analysis?	PN	No evidence of pre-specified subgroup analysis in protocol.
	5.2 ... multiple eligible outcome measurements (e.g. scales, definitions, time points) within the outcome domain?	N	No evidence of multiple eligible outcome measurements.
	5.3 ... multiple eligible analyses of the data?	N	Recognized outcome analysis.
	**Risk of bias judgement**	**Some concerns**	
**Overall bias**	**Risk of bias judgement**	**Some concerns**	

Year	2021	Study ID	TOURMALINE-MM2
**Domain**	**Signalling question**	**Response**	**Comments**
**Bias arising from the**	1.1 Was the allocation sequence random?	Y	A centralized randomization using IXRS was employed; double blinding.
	1.2 Was the allocation sequence concealed until participants were enrolled and assigned to interventions?	Y	

randomization process	1.3 Did baseline differences between intervention groups suggest a problem with the randomization process?	N	Baseline demographic and disease characteristics were well balanced between arms.
	**Risk of bias judgement**	**Low**	
**Bias due to deviations from intended interventions**	2.1.Were participants aware of their assigned intervention during the trial?	N	Quadruple masking (participant, care provider, investigator, outcomes assessor).
	2.2.Were carers and people delivering the interventions aware of participants' assigned intervention during the trial?	N	
	2.3. If Y/PY/NI to 2.1 or 2.2: Were there deviations from the intended intervention that arose because of the experimental context?	*NA*	
	2.4 If Y/PY to 2.3: Were these deviations likely to have affected the outcome?	*NA*	
	2.5. If Y/PY/NI to 2.4: Were these deviations from intended intervention balanced between groups?	*NA*	
	2.6 Was an appropriate analysis used to estimate the effect of assignment to intervention?	Y	Based on ITT.
	2.7 If N/PN/NI to 2.6: Was there potential for a substantial impact (on the result) of the failure to analyse participants in the group to which they were randomized?	*NA*	
	**Risk of bias judgement**	**Low**	
**Bias due to missing outcome data**	3.1 Were data for this outcome available for all, or nearly all, participants randomized?	PN	Over 5% of the patients in both groups were not evaluable.
	3.2 If N/PN/NI to 3.1: Is there evidence that result was not biased by missing outcome data?	PN	No evidence of correction of bias or sensitivity analysis.
	3.3 If N/PN to 3.2: Could missingness in the outcome depend on its true value?	PY	Patients with poor health status were more likely to lose follow up.
	3.4 If Y/PY/NI to 3.3: Is it likely that missingness in the outcome depended on its true value?	PN	
	**Risk of bias judgement**	**Some concerns**	
**Bias in measurement of the outcome**	4.1 Was the method of measuring the outcome inappropriate?	N	Response and disease progression assessments were based on central laboratory results and IMWG 2011 criteria.
	4.2 Could measurement or ascertainment of the outcome have differed between intervention groups?	N	Same measurements.
	4.3 Were outcome assessors aware of the intervention received by study participants?	N	Quadruple masking.
	4.4 If Y/PY/NI to 4.3: Could assessment of the outcome have been influenced by knowledge of intervention received?	*NA*	
	4.5 If Y/PY/NI to 4.4: Is it likely that assessment of the outcome was influenced by knowledge of intervention received?	*NA*	
	**Risk of bias judgement**	**Low**	
**Bias in selection of the reported result**	5.1 Were the data that produced this result analysed in accordance with a pre-specified analysis plan that was finalized before unblinded outcome data were available for analysis?	Y	Conformed with protocol.
	5.2 ... multiple eligible outcome measurements (e.g. scales, definitions, time points) within the outcome domain?	N	Recognized outcome measurements.
	5.3 ... multiple eligible analyses of the data?	N	Recognized outcome analysis.
	**Risk of bias judgement**	**Low**	
**Overall bias**	**Risk of bias judgement**	**Some concerns**	

**Year**	2021 **Study ID** MAIA		
**Domain**	**Signalling question**	**Response**	**Comments**
**Bias arising from the randomization process**	1.1 Was the allocation sequence random?	Y	Eligible patients were randomly assigned (1:1) using randomly permuted blocks (block size 4), by an interactive web response system.
	1.2 Was the allocation sequence concealed until participants were enrolled and assigned to interventions?	Y	The investigators or designated research staff enrolled and randomly assigned the patients. These individuals were further involved in the trial as part of the study team.
	1.3 Did baseline differences between intervention groups suggest a problem with the randomization process?	N	The baseline of included patients were balanced.
	**Risk of bias judgement**	**Low**	
	2.1.Were participants aware of their assigned intervention during the trial?	Y	Quadruple masking (participant, care provider, investigator, outcomes assessor).

**Bias due to deviations from intended interventions**	2.2.Were carers and people delivering the interventions aware of participants' assigned intervention during the trial?	Y	There was no masking to treatment assignments.
	2.3. If Y/PY/NI to 2.1 or 2.2: Were there deviations from the intended intervention that arose because of the experimental context?	N	No evidence of deviations.
	2.4 If Y/PY to 2.3: Were these deviations likely to have affected the outcome?	*NA*	
	2.5. If Y/PY/NI to 2.4: Were these deviations from intended intervention balanced between groups?	*NA*	
	2.6 Was an appropriate analysis used to estimate the effect of assignment to intervention?	N	The analysis of the effect of assignment to intervention was based on IMWG criteria.
	2.7 If N/PN/NI to 2.6: Was there potential for a substantial impact (on the result) of the failure to analyse participants in the group to which they were randomized?	N	No evidence of substantial impact.
	**Risk of bias judgement**	**Some concerns**	
**Bias due to missing outcome data**	3.1 Were data for this outcome available for all, or nearly all, participants randomized?	Y	All patients can be assessed by this outcome.
	3.2 If N/PN/NI to 3.1: Is there evidence that result was not biased by missing outcome data?	*NA*	
	3.3 If N/PN to 3.2: Could missingness in the outcome depend on its true value?	*NA*	
	3.4 If Y/PY/NI to 3.3: Is it likely that missingness in the outcome depended on its true value?	*NA*	
	**Risk of bias judgement**	**Low**	
**Bias in measurement of the outcome**	4.1 Was the method of measuring the outcome inappropriate?	N	The primary endpoint was progression-free survival (time from the date of randomization to either progressive disease or death, whichever occurred first), which was centrally assessed.
	4.2 Could measurement or ascertainment of the outcome have differed between intervention groups?	N	The two groups used the same outcome measurement.
	4.3 Were outcome assessors aware of the intervention received by study participants?	Y	No masking.
	4.4 If Y/PY/NI to 4.3: Could assessment of the outcome have been influenced by knowledge of intervention received?	N	The outcomes had recognized standards of assessment.
	4.5 If Y/PY/NI to 4.4: Is it likely that assessment of the outcome was influenced by knowledge of intervention received?	*NA*	
	**Risk of bias judgement**	**Low**	
**Bias in selection of the reported result**	5.1 Were the data that produced this result analysed in accordance with a pre-specified analysis plan that was finalized before unblinded outcome data were available for analysis?	Y	The analysis was in accordance with a pre-specified analysis plan.
	5.2 ... multiple eligible outcome measurements (e.g. scales, definitions, time points) within the outcome domain?	N	The measurement of the outcomes had a unified criteria.
	5.3 ... multiple eligible analyses of the data?	N	No signs of multiple eligible analyses of the data.
	**Risk of bias judgement**	**Low**	
**Overall bias**	**Risk of bias judgement**	**Some concerns**	

**Year**	2023 **Study ID** ATLAS		
**Domain**	**Signalling question**	**Response**	**Comments**
**Bias arising from the randomization process**	1.1 Was the allocation sequence random?	Y	A web-based system was used for randomization; no masking.
	1.2 Was the allocation sequence concealed until participants were enrolled and assigned to interventions?	PY	
	1.3 Did baseline differences between intervention groups suggest a problem with the randomization process?	N	Balanced baseline.
	**Risk of bias judgement**	**Low**	
**Bias due to deviations from intended interventions**	2.1.Were participants aware of their assigned intervention during the trial?	Y	Open-label trial.
	2.2.Were carers and people delivering the interventions aware of participants' assigned intervention during the trial?	Y	
	2.3. If Y/PY/NI to 2.1 or 2.2: Were there deviations from the intended intervention that arose because of the experimental context?	NI	Not reported.
	2.4 If Y/PY to 2.3: Were these deviations likely to have affected the outcome?	*NA*	

	2.5. If Y/PY/NI to 2.4: Were these deviations from intended intervention balanced between groups?	*NA*	
	2.6 Was an appropriate analysis used to estimate the effect of assignment to intervention?	Y	Based on ITT.
	2.7 If N/PN/NI to 2.6: Was there potential for a substantial impact (on the result) of the failure to analyse participants in the group to which they were randomized?	*NA*	
	**Risk of bias judgement**	**Some concerns**	
**Bias due to missing outcome data**	3.1 Were data for this outcome available for all, or nearly all, participants randomized?	Y	Over 95% of pts were evaluated.
	3.2 If N/PN/NI to 3.1: Is there evidence that result was not biased by missing outcome data?	*NA*	
	3.3 If N/PN to 3.2: Could missingness in the outcome depend on its true value?	*NA*	
	3.4 If Y/PY/NI to 3.3: Is it likely that missingness in the outcome depended on its true value?	*NA*	
	**Risk of bias judgement**	**Low**	
**Bias in measurement of the outcome**	4.1 Was the method of measuring the outcome inappropriate?	N	Measured according to the IMWG criteria.
	4.2 Could measurement or ascertainment of the outcome have differed between intervention groups?	N	Same measurement.
	4.3 Were outcome assessors aware of the intervention received by study participants?	Y	No masking.
	4.4 If Y/PY/NI to 4.3: Could assessment of the outcome have been influenced by knowledge of intervention received?	PN	The assessment of the outcomes depended primarily on objective laboratory results.
	4.5 If Y/PY/NI to 4.4: Is it likely that assessment of the outcome was influenced by knowledge of intervention received?	*NA*	
	**Risk of bias judgement**	**Low**	
**Bias in selection of the reported result**	5.1 Were the data that produced this result analysed in accordance with a pre-specified analysis plan that was finalized before unblinded outcome data were available for analysis?	PN	Outcomes reported as an unplanned interim analysis.
	5.2 ... multiple eligible outcome measurements (e.g. scales, definitions, time points) within the outcome domain?	N	No evidence of multiple eligible outcome measurements.
	5.3 ... multiple eligible analyses of the data?	N	No evidence of multiple eligible outcome analysis.
	**Risk of bias judgement**	**Some concerns**	
**Overall bias**	**Risk of bias judgement**	**Some concerns**	

**Year**	2021 **Study ID** RV-MM-PI-0752		
**Domain**	**Signalling question**	**Response**	**Comments**
**Bias arising from the randomization process**	1.1 Was the allocation sequence random?	NI	No detailed randomization plan provided.
	1.2 Was the allocation sequence concealed until participants were enrolled and assigned to interventions?	NI	
	1.3 Did baseline differences between intervention groups suggest a problem with the randomization process?	PN	Balanced baseline characteristics.
	**Risk of bias judgement**	**Some concerns**	
**Bias due to deviations from intended interventions**	2.1.Were participants aware of their assigned intervention during the trial?	Y	No masking.
	2.2.Were carers and people delivering the interventions aware of participants' assigned intervention during the trial?	Y	
	2.3. If Y/PY/NI to 2.1 or 2.2: Were there deviations from the intended intervention that arose because of the experimental context?	PN	No evidence of deviations.
	2.4 If Y/PY to 2.3: Were these deviations likely to have affected the outcome?	*NA*	
	2.5. If Y/PY/NI to 2.4: Were these deviations from intended intervention balanced between groups?	*NA*	
	2.6 Was an appropriate analysis used to estimate the effect of assignment to intervention?	Y	The effect of assignment to intervention was accessed according IMWG criteria.
	2.7 If N/PN/NI to 2.6: Was there potential for a substantial impact (on the result) of the failure to analyse participants in the group to which they were randomized?	*NA*	
	**Risk of bias judgement**	**Low**	

Bias due to missing outcome data	3.1 Were data for this outcome available for all, or nearly all, participants randomized?	Y	All patients can be assessed for progression-free survival.
	3.2 If N/PN/NI to 3.1: Is there evidence that result was not biased by missing outcome data?	*NA*	
	3.3 If N/PN to 3.2: Could missingness in the outcome depend on its true value?	*NA*	
	3.4 If Y/PY/NI to 3.3: Is it likely that missingness in the outcome depended on its true value?	*NA*	
	**Risk of bias judgement**	**Low**	
Bias in measurement of the outcome	4.1 Was the method of measuring the outcome inappropriate?	N	PFS was calculated from the time of enrollment until the date of PD or death resulting from any cause, whichever came first.
	4.2 Could measurement or ascertainment of the outcome have differed between intervention groups?	N	The measurement was same between intervention groups.
	4.3 Were outcome assessors aware of the intervention received by study participants?	Y	No masking.
	4.4 If Y/PY/NI to 4.3: Could assessment of the outcome have been influenced by knowledge of intervention received?	N	The assessment of the outcome had standard criteria.
	4.5 If Y/PY/NI to 4.4: Is it likely that assessment of the outcome was influenced by knowledge of intervention received?	*NA*	
	**Risk of bias judgement**	**Low**	
Bias in selection of the reported result	5.1 Were the data that produced this result analysed in accordance with a pre-specified analysis plan that was finalized before unblinded outcome data were available for analysis?	NI	No specific analysis plan in the protocol online.
	5.2 ... multiple eligible outcome measurements (e.g. scales, definitions, time points) within the outcome domain?	N	The measurement of the outcome had a unified concensus.
	5.3 ... multiple eligible analyses of the data?	N	No evidence of multiple eligible of the data.
	**Risk of bias judgement**	**Some concerns**	
**Overall bias**	**Risk of bias judgement**	**Some concerns**	

**Year**	2022 **Study ID** GRIFFIN		
**Domain**	**Signalling question**	**Response**	**Comments**
**Bias arising from the randomization process**	1.1 Was the allocation sequence random?	Y	A computer-generated randomization schedule was used under the supervision of the sponsor.
	1.2 Was the allocation sequence concealed until participants were enrolled and assigned to interventions?	Y	
	1.3 Did baseline differences between intervention groups suggest a problem with the randomization process?	N	Balanced baseline.
	**Risk of bias judgement**	**Low**	
**Bias due to deviations from intended interventions**	2.1.Were participants aware of their assigned intervention during the trial?	Y	Open-label trial.
	2.2.Were carers and people delivering the interventions aware of participants' assigned intervention during the trial?	Y	
	2.3. If Y/PY/NI to 2.1 or 2.2: Were there deviations from the intended intervention that arose because of the experimental context?	NI	Not reported.
	2.4 If Y/PY to 2.3: Were these deviations likely to have affected the outcome?	*NA*	
	2.5. If Y/PY/NI to 2.4: Were these deviations from intended intervention balanced between groups?	*NA*	
	2.6 Was an appropriate analysis used to estimate the effect of assignment to intervention?	Y	Based on ITT.
	2.7 If N/PN/NI to 2.6: Was there potential for a substantial impact (on the result) of the failure to analyse participants in the group to which they were randomized?	*NA*	
	**Risk of bias judgement**	**Some concerns**	
**Bias due to missing outcome data**	3.1 Were data for this outcome available for all, or nearly all, participants randomized?	Y	More than 95% pts were available.
	3.2 If N/PN/NI to 3.1: Is there evidence that result was not biased by missing outcome data?	*NA*	
	3.3 If N/PN to 3.2: Could missingness in the outcome depend on its true value?	*NA*	
	3.4 If Y/PY/NI to 3.3: Is it likely that missingness in the outcome depended on its true value?	*NA*	
	**Risk of bias judgement**	**Low**	

Bias in measurement of the outcome	4.1 Was the method of measuring the outcome inappropriate?	N	Outcomes were assessed by a validated computer algorithm in accordance with IMWG criteria.
	4.2 Could measurement or ascertainment of the outcome have differed between intervention groups?	N	Same assessments.
	4.3 Were outcome assessors aware of the intervention received by study participants?	Y	No blinding.
	4.4 If Y/PY/NI to 4.3: Could assessment of the outcome have been influenced by knowledge of intervention received?	N	The assessment of the outcomes depended primarily on objective laboratory findings.
	4.5 If Y/PY/NI to 4.4: Is it likely that assessment of the outcome was influenced by knowledge of intervention received?	*NA*	
	**Risk of bias judgement**	**Low**	
Bias in selection of the reported result	5.1 Were the data that produced this result analysed in accordance with a pre-specified analysis plan that was finalized before unblinded outcome data were available for analysis?	Y	Consistent with the pre-specified analysis plan.
	5.2 ... multiple eligible outcome measurements (e.g. scales, definitions, time points) within the outcome domain?	N	All eligible reported results for the outcome domain correspond to all intended outcome measurements.
	5.3 ... multiple eligible analyses of the data?	N	All eligible reported results for the outcome domain correspond to all intended outcome analysis.
	**Risk of bias judgement**	**Low**	
**Overall bias**	**Risk of bias judgement**	**Some concerns**	

**Year**	2023 **Study ID** GEM2014MAIN		
**Domain**	**Signalling question**	**Response**	**Comments**
**Bias arising from the randomization process**	1.1 Was the allocation sequence random?	NI	The only information about randomization methods is a statement that the study is randomized.
	1.2 Was the allocation sequence concealed until participants were enrolled and assigned to interventions?	NI	
	1.3 Did baseline differences between intervention groups suggest a problem with the randomization process?	N	Baseline characteristics were well balanced.
	**Risk of bias judgement**	**Some concerns**	
**Bias due to deviations from intended interventions**	2.1.Were participants aware of their assigned intervention during the trial?	Y	Open-label trial.
	2.2.Were carers and people delivering the interventions aware of participants' assigned intervention during the trial?	Y	
	2.3. If Y/PY/NI to 2.1 or 2.2: Were there deviations from the intended intervention that arose because of the experimental context?	NI	Not reported.
	2.4 If Y/PY to 2.3: Were these deviations likely to have affected the outcome?	*NA*	
	2.5. If Y/PY/NI to 2.4: Were these deviations from intended intervention balanced between groups?	*NA*	
	2.6 Was an appropriate analysis used to estimate the effect of assignment to intervention?	Y	Based on ITT.
	2.7 If N/PN/NI to 2.6: Was there potential for a substantial impact (on the result) of the failure to analyse participants in the group to which they were randomized?	*NA*	
	**Risk of bias judgement**	**Some concerns**	
**Bias due to missing outcome data**	3.1 Were data for this outcome available for all, or nearly all, participants randomized?	Y	No more than 5% pts were missing.
	3.2 If N/PN/NI to 3.1: Is there evidence that result was not biased by missing outcome data?	*NA*	
	3.3 If N/PN to 3.2: Could missingness in the outcome depend on its true value?	*NA*	
	3.4 If Y/PY/NI to 3.3: Is it likely that missingness in the outcome depended on its true value?	*NA*	
	**Risk of bias judgement**	**Low**	
**Bias in measurement of the outcome**	4.1 Was the method of measuring the outcome inappropriate?	N	Response and progression were assessed according to the International Myeloma Working Group criteria.
	4.2 Could measurement or ascertainment of the outcome have differed between intervention groups?	N	Same measurement.

	4.3 Were outcome assessors aware of the intervention received by study participants?	PY	Open-label trial.
	4.4 If Y/PY/NI to 4.3: Could assessment of the outcome have been influenced by knowledge of intervention received?	N	Assessment of the outcome needed to be supported by objective laboratory findings.
	4.5 If Y/PY/NI to 4.4: Is it likely that assessment of the outcome was influenced by knowledge of intervention received?	*NA*	
	**Risk of bias judgement**	**Low**	
**Bias in selection of the reported result**	5.1 Were the data that produced this result analysed in accordance with a pre-specified analysis plan that was finalized before unblinded outcome data were available for analysis?	Y	Consistent with the pre-specified analysis plan.
	5.2 ... multiple eligible outcome measurements (e.g. scales, definitions, time points) within the outcome domain?	N	Few possible ways in which the outcome domain can be measured.
	5.3 ... multiple eligible analyses of the data?	N	All eligible reported results for the outcome measurement correspond to all intended analyses.
	**Risk of bias judgement**	**Low**	
**Overall bias**	**Risk of bias judgement**	**Some concerns**	

**Year**	2023 **Study ID** PERSEUS		
**Domain**	**Signalling question**	**Response**	**Comments**
**Bias arising from the randomization process**	1.1 Was the allocation sequence random?	NI	The only information about randomization methods is a statement that the study is randomized.
	1.2 Was the allocation sequence concealed until participants were enrolled and assigned to interventions?	NI	
	1.3 Did baseline differences between intervention groups suggest a problem with the randomization process?	N	Baseline characteristics were well balanced.
	**Risk of bias judgement**	**Some concerns**	
**Bias due to deviations from intended interventions**	2.1.Were participants aware of their assigned intervention during the trial?	Y	Open-label trial.
	2.2.Were carers and people delivering the interventions aware of participants' assigned intervention during the trial?	Y	
	2.3. If Y/PY/NI to 2.1 or 2.2: Were there deviations from the intended intervention that arose because of the experimental context?	NI	Not reported.
	2.4 If Y/PY to 2.3: Were these deviations likely to have affected the outcome?	*NA*	
	2.5. If Y/PY/NI to 2.4: Were these deviations from intended intervention balanced between groups?	*NA*	
	2.6 Was an appropriate analysis used to estimate the effect of assignment to intervention?	Y	Based on ITT.
	2.7 If N/PN/NI to 2.6: Was there potential for a substantial impact (on the result) of the failure to analyse participants in the group to which they were randomized?	*NA*	
	**Risk of bias judgement**	**Some concerns**	
**Bias due to missing outcome data**	3.1 Were data for this outcome available for all, or nearly all, participants randomized?	Y	All patients can be assessed by this outcome.
	3.2 If N/PN/NI to 3.1: Is there evidence that result was not biased by missing outcome data?	*NA*	
	3.3 If N/PN to 3.2: Could missingness in the outcome depend on its true value?	*NA*	
	3.4 If Y/PY/NI to 3.3: Is it likely that missingness in the outcome depended on its true value?	*NA*	
	**Risk of bias judgement**	**Low**	
**Bias in measurement of the outcome**	4.1 Was the method of measuring the outcome inappropriate?	N	Measured according to the IMWG criteria.
	4.2 Could measurement or ascertainment of the outcome have differed between intervention groups?	N	Same measurement.
	4.3 Were outcome assessors aware of the intervention received by study participants?	Y	No masking.
	4.4 If Y/PY/NI to 4.3: Could assessment of the outcome have been influenced by knowledge of intervention received?	PN	Assessment of the outcome needed to be supported by objective laboratory findings.
	4.5 If Y/PY/NI to 4.4: Is it likely that assessment of the outcome was influenced by knowledge of intervention received?	*NA*	

	**Risk of bias judgement**	**Low**	
**Bias in selection of the reported result**	5.1 Were the data that produced this result analysed in accordance with a pre-specified analysis plan that was finalized before unblinded outcome data were available for analysis?	Y	The analysis was in accordance with a pre-specified analysis plan.
	5.2 ... multiple eligible outcome measurements (e.g. scales, definitions, time points) within the outcome domain?	N	The measurement of the outcomes had unified criteria.
	5.3 ... multiple eligible analyses of the data?	N	No signs of multiple eligible analyses of the data.
	**Risk of bias judgement**	**Low**	
**Overall bias**	**Risk of bias judgement**	**Some concerns**	

References

1. Gelman A, Rubin DB. Markov chain Monte Carlo methods in biostatistics. Stat Methods Med Res. 1996;5(4):339-55.

2. Brooks SP, Gelman A. General Methods for Monitoring Convergence of Iterative Simulations. Journal of Computational and Graphical Statistics. 1998;7(4):434-455.

3. Higgins JP, Thompson SG. Quantifying heterogeneity in a meta-analysis. Stat Med. 2002;21(11):1539-58.

4. Dias S, Welton NJ, Caldwell DM, Ades AE. Checking consistency in mixed treatment comparison meta-analysis. Stat Med. 2010;29(7-8):932-44.

5. Mikhael JR, Dingli D, Roy V, Reeder CB, Buadi FK, Hayman SR, et al. Management of newly diagnosed symptomatic multiple myeloma: updated Mayo Stratification of Myeloma and Risk-Adapted Therapy (mSMART) consensus guidelines 2013. Mayo Clin Proc. 2013;88(4):360-76.

6. Durie BG, Salmon SE. A clinical staging system for multiple myeloma. Correlation of measured myeloma cell mass with presenting clinical features, response to treatment, and survival. Cancer. 1975;36(3):842-54.

7. Greipp PR, San Miguel J, Durie BG, Crowley JJ, Barlogie B, Bladé J, et al. International staging system for multiple myeloma. J Clin Oncol. 2005;23(15):3412-20.

8. Palumbo A, Avet-Loiseau H, Oliva S, Lokhorst HM, Goldschmidt H, Rosinol L, et al. Revised International Staging System for Multiple Myeloma: A Report From International Myeloma Working Group. J Clin Oncol. 2015;33(26):2863-9.

9. Attal M, Lauwers-Cances V, Marit G, Caillot D, Moreau P, Facon T, et al. Lenalidomide maintenance after stem-cell transplantation for multiple myeloma. N Engl J Med. 2012;366(19):1782-91.

10. Dimopoulos MA, Gay F, Schjesvold F, Beksac M, Hajek R, Weisel KC, et al. Oral ixazomib maintenance following autologous stem cell transplantation (TOURMALINE-MM3): a double-blind, randomised, placebo-controlled phase 3 trial. Lancet. 2019;393(10168):253-264.

11. Jackson GH, Davies FE, Pawlyn C, Cairns DA, Striha A, Collett C, et al. Lenalidomide maintenance versus observation for patients with newly diagnosed multiple myeloma (Myeloma XI): a multicentre, open-label, randomised, phase 3 trial. Lancet Oncol. 2019;20(1):57-73.

12. Facon T, Dimopoulos MA, Dispenzieri A, Catalano JV, Belch A, Cavo M, et al. Final analysis of survival outcomes in the phase 3 FIRST trial of up-front treatment for multiple myeloma. Blood. 2018;131(3):301-310.

13. Mateos MV, Cavo M, Blade J, Dimopoulos MA, Suzuki K, Jakubowiak A, et al. Overall survival with daratumumab, bortezomib, melphalan, and prednisone in newly diagnosed multiple myeloma (ALCYONE): a randomised, open-label, phase 3 trial. Lancet. 2020;395(10218):132-141.

14. Bringhen S, D'Agostino M, Paris L, Ballanti S, Pescosta N, Spada S, et al. Lenalidomide-based induction and maintenance in elderly newly diagnosed multiple myeloma patients: updated results of the EMN01 randomized trial. Haematologica. 2020;105(7):1937-1947.

15. Zweegman S, Stege CAM, Haukas E, Schjesvold FH, Levin MD, Waage A, et al. Ixazomib-Thalidomide-low dose dexamethasone induction followed by maintenance therapy with ixazomib or placebo in newly diagnosed multiple myeloma patients not eligible for autologous stem cell transplantation; results from the randomized phase II HOVON-126/NMSG 21.13 trial. Haematologica. 2020;105(12):2879-2882.

16. Dimopoulos MA, Špička I, Quach H, Oriol A, Hájek R, Garg M, et al. Ixazomib as Postinduction Maintenance for Patients With Newly Diagnosed Multiple Myeloma Not Undergoing Autologous Stem Cell Transplantation: The Phase III TOURMALINE-MM4 Trial. J Clin Oncol. 2020;38(34):4030-4041.
17. Moreau P, Hulin C, Perrot A, Arnulf B, Belhadj K, Benboubker L, et al. Maintenance with daratumumab or observation following treatment with bortezomib, thalidomide, and dexamethasone with or without daratumumab and autologous stem-cell transplant in patients with newly diagnosed multiple myeloma (CASSIOPEIA): an open-label, randomised, phase 3 trial. Lancet Oncol. 2021;22(10):1378-1390.
18. Gay F, Musto P, Rota-Scalabrini D, Bertamini L, Belotti A, Galli M, et al. Carfilzomib with cyclophosphamide and dexamethasone or lenalidomide and dexamethasone plus autologous transplantation or carfilzomib plus lenalidomide and dexamethasone, followed by maintenance with carfilzomib plus lenalidomide or lenalidomide alone for patients with newly diagnosed multiple myeloma (FORTE): a randomised, open-label, phase 2 trial. Lancet Oncol. 2021;22(12):1705-1720.
19. Facon T, Venner CP, Bahlis NJ, Offner F, White DJ, Karlin L, et al. Oral ixazomib, lenalidomide, and dexamethasone for transplant-ineligible patients with newly diagnosed multiple myeloma. Blood. 2021;137(26):3616-3628.
20. Larocca A, Bonello F, Gaidano G, D'Agostino M, Offidani M, Cascavilla N, et al. Dose/schedule-adjusted Rd-R vs continuous Rd for elderly, intermediate-fit patients with newly diagnosed multiple myeloma. Blood. 2021;137(22):3027-3036.
21. Facon T, Kumar SK, Plesner T, Orlowski RZ, Moreau P, Bahlis N, et al. Daratumumab, lenalidomide, and dexamethasone versus lenalidomide and dexamethasone alone in newly diagnosed multiple myeloma (MAIA): overall survival results from a randomised, open-label, phase 3 trial. Lancet Oncol. 2021;22(11):1582-1596.
22. Kaufman JL, Laubach JP, Sborov D, Reeves B, Rodriguez C, Chari A, et al. Daratumumab (DARA) Plus Lenalidomide, Bortezomib, and Dexamethasone (RVd) in Patients with Transplant-Eligible Newly Diagnosed Multiple Myeloma (NDMM): Updated Analysis of Griffin after 12 Months of Maintenance Therapy. Blood. 2020;136(Suppl 1):45-46.
23. Dytfeld D, Wróbel T, Jamroziak K, Kubicki T, Robak P, Walter-Croneck A, et al. Carfilzomib, lenalidomide, and dexamethasone or lenalidomide alone as maintenance therapy after autologous stem-cell transplantation in patients with multiple myeloma (ATLAS): interim analysis of a randomised, open-label, phase 3 trial. Lancet Oncol. 2023;24(2):139-150.
24. Rosiñol, L.; Oriol, A.; Ríos, R.; Blanchard, M. J.; Jarque, I.; Bargay, J.; Hernández, M. T.; Cabañas, V.; Carrillo-Cruz, E.; Sureda, A., et al., Lenalidomide and dexamethasone maintenance with or without ixazomib, tailored by residual disease status in myeloma. Blood 2023, 142, (18), 1518-1528.
25. Sonneveld, P.; Dimopoulos, M. A.; Boccadoro, M.; Quach, H.; Ho, P. J.; Beksac, M.; Hulin, C.; Antonioli, E.; Leleu, X.; Mangiacavalli, S., et al., Daratumumab, Bortezomib, Lenalidomide, and Dexamethasone for Multiple Myeloma. N Engl J Med 2024, 390, (4), 301-313.
